# Recommendations for Quantitative Data-Independent Acquisition (DIA) Proteomics Using Controlled Quantitative Experiments (CQEs)

**DOI:** 10.1021/acs.jproteome.6c00328

**Published:** 2026-10-22

**Authors:** Andrew M. Frey, Frances Sidgwick, Andrew Porter, Pawel Palmowski, Matthias Trost

**Affiliations:** † Biosciences Institute, 5994 Newcastle University, Newcastle-upon-Tyne NE2 4HH, U.K.; ‡ Translational and Clinical Research Institute, Newcastle University, Newcastle-upon-Tyne NE2 4HH, U.K.; § Division of Molecular & Cellular Function, University of Manchester, Manchester M13 9NT, U.K.

**Keywords:** Proteomics, quantitation, accuracy, precision, label free quantification, LFQ, Data Independent Acquisition, DIA

## Abstract

Advances in mass spectrometry and associated computational tools have resulted in the widespread adoption of Data-Independent Acquisition (DIA) for proteomics. Experiments using state-of-the-art instrumentation and specialized modes of acquisition report ever-increasing throughput and protein identification rates. However, experiments to test the efficacy of protein quantitation in these novel methods are surprisingly rare, especially in novel methods where low sample input or high throughput is considered. Here, we performed a series of controlled quantitative experiments (CQEs) on two commonly used mass spectrometry platforms (Exploris480 and timsTOF HT), using defined mixtures of human, yeast, and bacterial proteomes. Peptide spectrum matching and initial quantification were performed using DIA-NN, with a series of postprocessing options also compared. Overall, we identified >10,000 unique proteins and >160,000 precursors across samples with excellent accuracy and precision. Additional postprocessing was found to have drastic impacts on ID rates, quantitative precision and accuracy, and false quantitative rate (FQR). These impacts were particularly notable in experiments using low amounts of sample. In optimal loading conditions, data processing could reduce FQR from ∼0.1 to 0% with minimal losses in ID rates. Ultimately, we present a series of recommendations for proteomics data processing and advice on the use of CQEs and resulting metrics when assessing quantitative efficacy in novel modes of acquisition.

## Graphical abstract

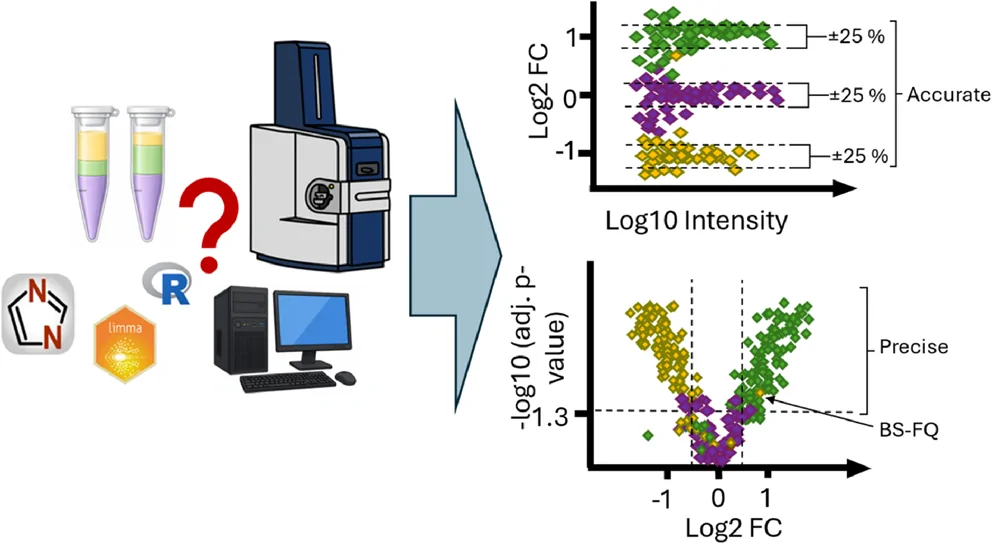

## Introduction

Quantitative proteomics has made substantial progress over the past decade, with mass spectrometry (MS) becoming a cornerstone of proteomic research.^1^ This development has cemented MS’s role as an essential tool in biological and biomedical studies. Despite these advancements, achieving efficacious quantitative analysis across the vast dynamic range of the proteome remains a major challenge. The proteome spans an immense range: the cellular proteome covers 7 orders of magnitude, while plasma proteomes extend over ten.^2,3^ Progress in the field has been propelled by innovations in hardware and data acquisition technologies, pushing proteomics toward more sophisticated applications, particularly in clinical settings for disease diagnostics and biomarker discovery.

In response to these demands, there has been a shift in proteomics from focusing on detecting as many peptides and proteins as possible to prioritising throughput and quantification,^4,5^ and a transition from mostly data dependent acquisition (DDA) to data-independent acquisition (DIA) strategies: DIA LC-MS/MS is now the cornerstone MS acquisition method utilized in proteomic analysis,^6,7^ with recent advancements in software tools and faster mass spectrometers driving its expansion.^8^ Innovations in MS instrumentation, particularly in the sensitivity and speed of mass analysers, and the adoption of shorter liquid chromatography (LC) gradients have contributed to the success of this approach.^9,10^ These combined with enhanced data analysis techniques have led to a preference for label-free quantification (LFQ) methods. LFQ offers multiple benefits, including reduced costs, fewer chemical requirements, and no sample number limitations.^11–13^ As the field moves toward high throughput analysis, the adoption of shorter LC gradients has become more common to reduce overall acquisition times. Shorter LC gradients lead to reduced peak widths, and the speed of the mass analyzer becomes crucial for generating enough data points per peak to ensure quantitative accuracy. Although longer LC gradients provide deeper proteome coverage and greater peptide identification, they come at the cost of increased analysis time.

Despite the (now routine) detection of thousands of proteins in recent studies, quantifying low-abundance proteins remains a challenge.^14^ Variations in false discovery rate (FDR) thresholds and quantifiable peak identification across studies complicate comparisons, emphasizing the need for rigorous benchmarking of technologies.^15,16^ Managing the FDR is a major statistical challenge in shotgun proteomics. The most common method for addressing this is target-decoy competition (TDC) at the peptide-spectrum match (PSM) level, where observed spectra are searched against a database containing both genuine (target) peptides and decoy peptides. At the peptide level, the top-scoring PSM for each peptide is selected, and FDR is estimated based on this information. In proteomics, a FDR threshold of 1% is commonly accepted, meaning that 1% of the quantifiable precursors will be false positives.

Reporting the total number of proteins identified is a standard output in proteomics experiments. However, relying solely on this metric can be misleading, as it may inflate results and fail to account for reproducibility within data sets. More proteins are typically identified than those that are quantifiable, and the total protein count does not reflect the consistency across replicates.^13,17^ Therefore, it is crucial to assess not only the number of identified proteins but also their quantifiability.

Data points per peak (PPP) is widely considered critical for reproducibility and accurate quantification, though the minimum required remains debated. Estimates range from six^18^ to eight,^19^ depending on whether peak shape definition or robust quantification is the goal, and on the quantitative metric used (peptide, precursor, or protein level). As workflows shift toward higher throughput, PPP tends to fall: shorter gradients narrow peaks, and larger DIA windows lengthen duty cycle times, together reducing the measurements available per peak. Yet lower PPP is not uniformly detrimental as reduced PPP can boost protein identifications while preserving quantitative precision.^20^ The practical impact likely depends on study design: minimal retention-time drift across consecutive injections may tolerate fewer points, whereas longer studies with greater retention-time shifts are more vulnerable to under-sampling.

Some potential drawbacks to excluding precursors based on PPP include introduction of missing values and reduced reproducibility across replicates. Furthermore, the number of data points and protein identifications alone does not indicate reproducibility within a data set. To address this, CV values are used to assess reproducibility. The CV expresses variance as a percentage of the mean, with higher values indicating greater dispersion around the mean. Lower CV values suggest good reproducibility, which is vital for ensuring the reliability of the data. But this also does not provide assurance of quantitative efficacy.

LFQ mixed proteome experiments were initially designed by Navarro et al. in 2016 ^13^ and are now widely utilized.^8,18,19^ These involve the analysis of multiple proteomes in a single experimental setup without the use of isotopic or chemical labels. Cell lysates from multiple organisms or conditions are first digested and peptides are then pooled together in a single sample, with the ratio of each proteome in the mixture being carefully controlled. Although the absolute concentration of individual proteins is not known, this experimental design allows quantification of different protein ratios for each organism and enables in-depth evaluation of precision and accuracy of the measurements, as well as the proportion of proteins in opposition to the ground truth.

In this manuscript, we describe a series of these controlled quantitative experiments (CQEs), including two- and three- proteome experiments. In addition to describing overall fold change distribution and variance, we calculate additional metrics and use these to interrogate a number of key parameters in MS-proteomics workflows. In addition to MS acquisition, postprocessing decisions-such as the number of peptides identified per protein, the precursor intensity and data points per peak-and the coefficient of variation (CV) across replicates were investigated as options to consider when filtering the acquired DIA data to ensure reliable quantitative results during LC-MS method design. Ultimately, we provide a series of recommendations for optimal data processing and best practice for assurance of quantitative efficacy in DIA-LFQ proteomics.

## Materials and Methods

### Species, Strains, and Culture Methods

HeLa cells were routinely cultured in Dulbecco’s Modified Eagle Medium (DMEM) with 10% (v/v) Foetal Calf Serum, and 15 mM l-Glutamine, and were passaged at ∼80% confluency. Prior to collection, cells were washed three times with ice cold PBS and detached by scraping. Cells were centrifuged (5 min, 1000*g*, 4 °C) and stored at −80 °C. *Escherichia coli* (DH5α) were cultured overnight in LB medium at 37 °C in an orbital shaker at 50 rpm. *Saccharomyces cerevisiae* was cultured overnight in Yeast-Peptone-Dextrose (YPD) broth at 37 °C in an orbital shaker at 50 rpm. Microbial liquid cultures were collected by centrifugation (10 min, 4000*g*, 4 °C), and washed three times with ice cold PBS.

### S-Trap Protein Digestion

Samples were lysed in 500 μL 5% SDS, 50 mM triethylammonium bicarbonate (TEAB). Following protein quantification (Pierce BCA assay, ThermoFisher Scientific) 1 mg of each sample was reduced by adding Tris­(2-carboxyethyl)­phosphine (TCEP) to a final concentration of 10 mM for 30 min at room temperature followed by alkylation with 20 mM iodoacetamide for 30 min in the dark. Samples were then subjected to suspension trapping and digestion with a commercially available kit (Protifi), according to manufacturer’s instructions. Briefly, samples were acidified by addition of 50 μL of 12% phosphoric acid and diluted with 3.3 mL of S-trap binding buffer: 90% MeOH, 100 mM TEAB, pH 7.1. The acidified samples were then loaded onto the S-trap midi spin column and centrifuged for 1 min at 4000*g*. Columns were washed five times with S-trap binding buffer before addition of porcine trypsin (1:10) (Worthington) in 350 μL of 50 mM TEAB to the column. Samples were incubated at 37 °C overnight. Peptides were eluted by washing the column with first 50 mM TEAB, pH 8.0 (500 μL), followed by 500 μL of 0.2% formic acid (FA) and finally 500 μL of 0.2% FA, 50% acetonitrile. Peptides were then dried under vacuum. Peptides from separate species were resuspended and combined in defined ratios as described in the Results section.

### E480 LC-MS Methods

Protein digests were dissolved in 0.2% trifluoroacetic acid and injected with an UltiMate 3000 RSLCnano System (Thermo Fisher). Samples were trapped on an Acclaim PepMap 100 C18 LC trap column (Inner diameter: 100 μm × 20 mm, 3 μm, 100 Å) and then separated on an 75 cm EASY-Spray nanoLC C18 column (Inner diameter: 75 μm × 500 mm, 2 μm, 100 Å; Thermo-Fisher) at a flow rate of 150 nL/min. Solvent A was water containing 0.1% formic acid, and solvent B was 80% acetonitrile containing 0.1% formic acid. The gradient used for analysis of samples was as follows: Solvent B was maintained at 3% for 5 min, followed by an increase from 3% to 35% B over 120 min, 35 to 90% B over 0.5 min, maintained at 90% B for 5 min, followed by a decrease to 3% over 0.5 min and equilibration at 3% for 25 min.

The Exploris 480 mass spectrometer was operated in positive ion, DIA mode. Full MS scan spectra were acquired in the *m*/*z* range of 350–1050 with normalized automatic gain control target of 1000%, and maximum injection time set to dynamic, at a resolution of 60,000. Targeted MS2 scan spectra were acquired using either 20, 76, or 152 isolation windows (Table S1). CE set to normalized if not stated. AGC target of 3000%, at a resolution of 30,000 with dynamic injection time. Electrospray voltage was static, and capillary temperature was 275 °C, with expected LC peak width of 30s. No sheath and auxiliary gas flow were used

### timsTOF-HT LC-TIMS-MS/MS Methods

LC was performed using an Evosep One system with a 15 cm Aurora Elite C18 column with integrated captive spray emitter (IonOpticks), at 50 °C. Buffer A was 0.1% formic acid in HPLC water, buffer B was 0.1% formic acid in acetonitrile. Immediately prior to LC-MS, peptides were resuspended in buffer A and a volume equivalent to 500 ng was loaded onto the LC system-specific C18 EvoTips, according to manufacturer instructions, and subjected to the predefined Whisper-Zoom 20 SPD protocol (where the gradient is 0–35% buffer B, 200 nL/min, for 58 min, 20 samples per day were permitted). For high-sensitivity experiments the Whisper-Zoom 40 SPD protocol (40 samples per day permitted) was used, with 250 pg of protein digest loaded. The Evosep One was used in line with a timsTOF-HT mass spectrometer (Bruker) operated in diaPASEF mode. Mass and IM ranges were 300–1200 *m*/*z* and 0.6–1.45 1/*K*_0_. diaPASEF was performed using 16 variable width IM-*m*/*z* windows each with two frames, without overlap (Table S2). TIMS ramp and accumulation times were varied as indicated in the results, though for the most frequently used 66 ms, total cycle time was ∼1.2 s. For high sensitivity, a 4-window scheme (Table S3) with 300 ms accumulation time was used. Collision energy was applied in a linear fashion, where ion mobility = 0.6–1.6 1/*K*_0_, and collision energy = 20–59 eV.

### Data Analysis

Raw files were searched using DIA-NN version 1.9.2 ^20^ against a Uniprot *H. sapiens*, *E. coli*, and *S.* *cerevisiae* reference database to identify protein groups (containing 30826 proteins-see Figure 3, downloaded July 2024) as well as a common contaminants database,^21^ at 1% false discovery rate with default settings and DIA-NN normalization selected, (with the exception that searches for the timsTOF-HT were set to precursors with *m*/*z* 300–1200). *In silico* digest for spectral library generation was used for library-free search. For high sensitivity (250 pg) experiments on the timsTOF-HT, an empirical DIA-spectral library was used. This was generated by subjecting 50 ng of matched samples (equivalent to “bulk” preparations in single-cell experiments) to the same LC-TIMS-MS high sensitivity protocol, analyzed by DIA-NN as described directly above. All resulting data sets underwent postprocessing as follows: The report.parquet DIA-NN output file was subjected to processing in Rstudio. Key packages included diannR, tidyverse, ggplot2, plotly, and dyplyr packages. Protein groups were assigned and quantified by MaxLFQ using report.parquet files and the diannR max_lfq function, including precursor and protein group Q-value filtering set to 0.01. Prior to this, the report.parquet was filtered using a number of possible options as described in the Results section, namely precursor CV filtering, precursor PPP filtering, and/or precursor percentile filtering. Protein Groups were filtered to remove proteins absent across all replicates and those with mean intensities falling below the first or above the 99th percentile. Filtered precursor intensities were then aggregated to protein groups using the MaxLFQ algorithm (diann_maxlfq) with DIA-NN Protein.Group annotations. Protein groups were subsequently required to be supported by at least 1–4 unique peptide sequences. Further details on processing parameters are described in the relevant Results section. Common to all analyses was the use of Limma for statistics,^22^ where *p*-values were adjusted using the Benjamini–Hochberg false discovery rate (FDR) method, and that genes meeting the specified adjusted *p*-value threshold (<0.05) were considered statistically significant. Also common to all analyses was the automated exclusion of contaminants, and only including proteins quantified in at least half (3/5 or 2/3) of replicates for both conditions in a given comparison.

## Results

### Optimizing DIA Parameters with Two-Species Controlled Quantitative Experiments (CQEs)

Controlled quantitative experiments (CQEs) provide a robust framework for evaluating both mass spectrometry acquisition parameters and subsequent data analysis workflows. To optimize our data-independent acquisition (DIA) strategy, we employed a two-species CQE using a tryptic digest mixture of human (HeLa) and bacterial (*E. coli*) proteins. The mixtures were prepared at two different ratios of human to bacterial proteins (1:2 and 2:1) while maintaining a constant total protein load.

The performance of the tested acquisition parameters was evaluated using metrics summarized in Table 1. Accuracy was quantified as the inverse false quantitative rate (inv-FQR, %) and defined as the proportion of proteins with fold changes within ±25% of the expected value (approximately 2σ), a stricter threshold than the ±50% commonly used in proteomics studies. It is however worth noting the suitability of a fold-change threshold depends on the study objectives, the technical variability, the expected biological variability, and the magnitude of biologically relevant changes. While stricter thresholds can increase confidence in quantitative accuracy, they may also exclude true positive findings and biologically meaningful changes with smaller effect sizes. Precision was assessed as the proportion of statistically significant hits. To address the risk of biologically misleading results, we additionally calculated the biologically significant false quantitative rate (BS-FQR, %), defined as the proportion of statistically significant proteins with fold changes in the incorrect direction.

**Table 1:** Description of CQE Metrics^a^

Measure	Metric	Description	Caveats^a^
Accuracy	Inv-FQR (%)	100 - (no. proteins outside a factor of ±0.25 ground truth/no. proteins in the proteome) × 100	• Factor of ±0.25 is related to (half of) the commonly used minimal fold change threshold of ±0.5.
Precision	Significant hits (%)	(no. proteins with significant hits/no. proteins in the proteome) × 100	• Cannot be applied to CQE conditions where a fold change of 1 is expected (i.e., no fold change).
			• See legend^b^
Misleading hits/false positives	BS-FQR (%)	(no. proteins with significant hits in the opposite direction to the ground truth/no. proteins in the proteome) × 100	• Where 1-fold change (i.e., no fold change) is expected, a cutoff of ±1.5 FC (∼2σ) can be applied.
			• See legend^b^

a A protein group is falsely quantified (FQ) if relative abundance falls outside of ±25% of the ground-truth, with the proportion of FQ proteins within the proteome deriving the false quantitative rate (FQR, %). The inverse of this (inv-FQR) facilitates reporting of accuracy since optimal accuracy would be no proteins falsely quantified, i.e., FQR 0% and inv-FQR 100%. The biologically significant-false quantitative rate (BS-FQR, %) is defined as the proportion of protein groups with fold-changes that are statistically significant (here, by *t* test with FDR-adjusted *P*-value <0.05), and with a fold-change in the opposite direction to that of the ground truth (i.e., protein groups with an increase in relative abundance when a decrease is expected, or vice versa). When no fold change is expected, a cutoff of ±1.5 FC can be applied for BS-FQR calculation. From an analytical standpoint, the inv-FQR and significant hits provide the most critical insights into quantitative accuracy and precision, while BS-FQR is important biologically, since it relates to protein groups that are contrary to the ground truth and are inferred to be statistically significant.

b Metrics are partially dependent on the ground truth i.e., the expected fold change. Using these metrics, it is anticipated that BS-FQR negatively-, and precision positively correlates with expected fold change. Metrics are also partially dependent on the statistical test used and the number of replicates. These features are useful, as they permit assessment of precision and the likelihood of misleading hits at a given fold change, and by using these metrics the CQEs also assess choice of statistical analysis and number of replicates, which are essential parameters of experimental design.

Given the known composition of the mixtures, the optimal outcome would be characterized by human proteins with log_2_ fold changes close to +1 and bacterial proteins close to −1, with no proteins exceeding the range of 1.5–2.5-fold change in the correct direction (corresponding to 100% inv-FQR). In such an ideal case, all proteins would be statistically significant (100% significant hits) with a BS-FQR of 0%, and the number of quantified protein groups would be maximized.

#### Recommendation: Perform Two-Species CQEs for DIA Parameter Optimization

When testing DIA acquisition parameters, a simple two-species CQE provides an effective means to assess quantitative performance while simultaneously capturing information on identification rates. In this study, human–bacterial protein mixtures were analyzed using a 20 SPD (58 min active gradient) method on the Evosep One coupled to a timsTOF HT operating in diaPASEF mode. MS1- and MS2-PASEF settings (16 windows, 2 frames) were held constant, while the trapped ion mobility spectrometry (TIMS) accumulation and ramp times (TART) were varied between 33, 66, and 100 ms, corresponding to duty cycles of approximately 0.6, 1.2, and 1.8 s, respectively. Protein digest loads of 50, 100, 500, and 1000 ng were tested (Figure 1 and Figure S1). Postprocessing involved filtering for proteins identified by at least two peptides per protein group, exclusion of putative contaminants, and statistical analysis using *limma*.^22^ The full output resulting from the analysis is provided (Table S4).

**Figure 1:**
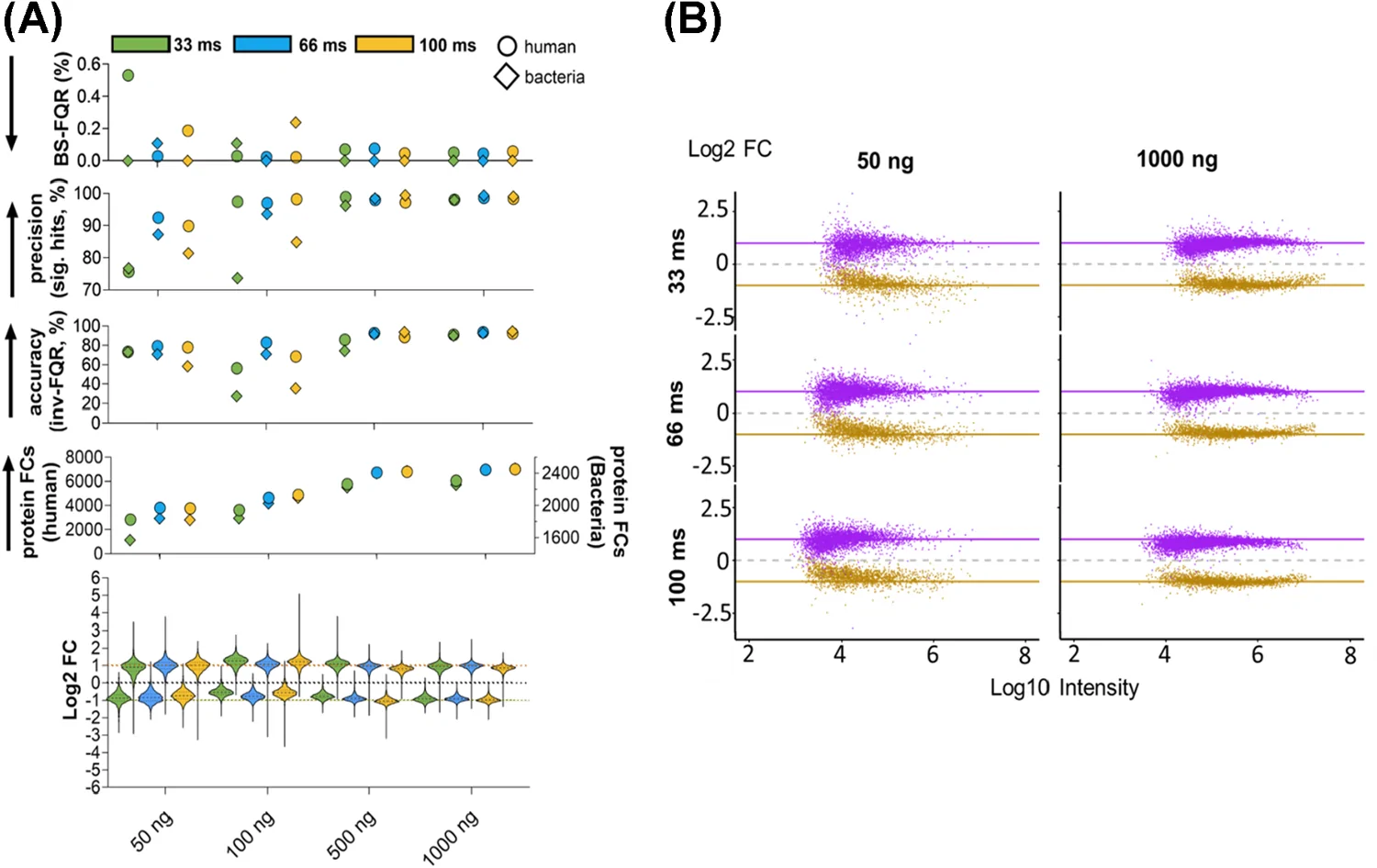
Initial assessment of quantitative efficacy for diaPASEF with variable TART and load. (A) Summary of metrics for TART and load conditions, lower to upper figures; Log2 FC (median, 25% quartiles, outliers shown, expected FC and zero are indicated by dashed lines), protein group FCs, Significant hits, FQR, and BS-FQR are shown. Arrows indicate the preferable result vector for each metric, i.e., If a higher result is better (arrow pointing up) or worse (arrow pointing down). (B) Distribution of (log10) protein group intensities against (log2) fold change for all TART, highest and lowest load (50 and 1000 ng). Purple = Human, Khaki = Bacteria. Data shown are derived from three replicates.

At the highest load (1000 ng), approximately 10,000 unique proteins and 100,000–140,000 precursors were identified, with an average of 3.5–5.5 MS2 points per peak at full width half-maximum (PPP-FWHM; Figure S1). Across all TART and load combinations, quantitation was robust (Figure 1A), with median fold changes of 0.97 ± 0.09 for human proteins and −0.80 ± 0.10 for bacterial proteins. Precursor and protein identifications increased with TART, whereas PPP-FWHM and MS2 signal decreased. Lower-abundance proteins were quantified with reduced precision and accuracy, as expected, and both metrics declined further with decreasing protein load (Figure 1A). Notably, very highly abundant proteins were less well quantified at higher TART values (Figure 1B), possibly due to space-charge effects within the TIMS cell or detector saturation.

Overall, the majority of quantitative metrics (Figure 1) indicated that a TART of 66 ms with a 1000 ng protein load offered the best performance, with minimal loss in total identifications compared to a 100 ms TART. Median normalization distorted log_2_ fold changes from the known ground truth (Figure S2) and was therefore deemed unsuitable for this data set.

### Assessing Quantitative Efficacy across the Entire Proteomics Pipeline Using Complex Proteomes

The two-species CQEs described above evaluate quantitative efficacy under conditions where most proteins change modestly in abundance. As a result, conclusions drawn from these experiments may not be generalizable to scenarios involving more substantial proteome-wide changes or indeed more typical experiments where the majority of the proteome remains the same. To address this, we designed an additional series of CQEs using three-species mixtures comprising human (HeLa), yeast (*S. cerevisiae*), and bacterial (*E. coli*) proteins (Figure 2A). The mixtures were prepared in defined ratios to produce large, modest, or small abundance changes affecting different proportions of the proteome. This design reflects a range of possible outcomes in biological experiments, enabling assessment of quantitative efficacy across varied scenarios and providing limited opportunities for false discovery rate (FDR) evaluation. Comparisons 1, 2, and 5 represent cases where only a small proportion of the proteome undergoes modest changes, which is most typical in biological studies. In contrast, comparisons 3, 4, and 6 represent extreme conditions where most or all proteins exhibit substantial abundance changes, as might occur when analyzing distinct tissues, organelle-enriched fractions, or markedly divergent physiological states.

**Figure 2:**
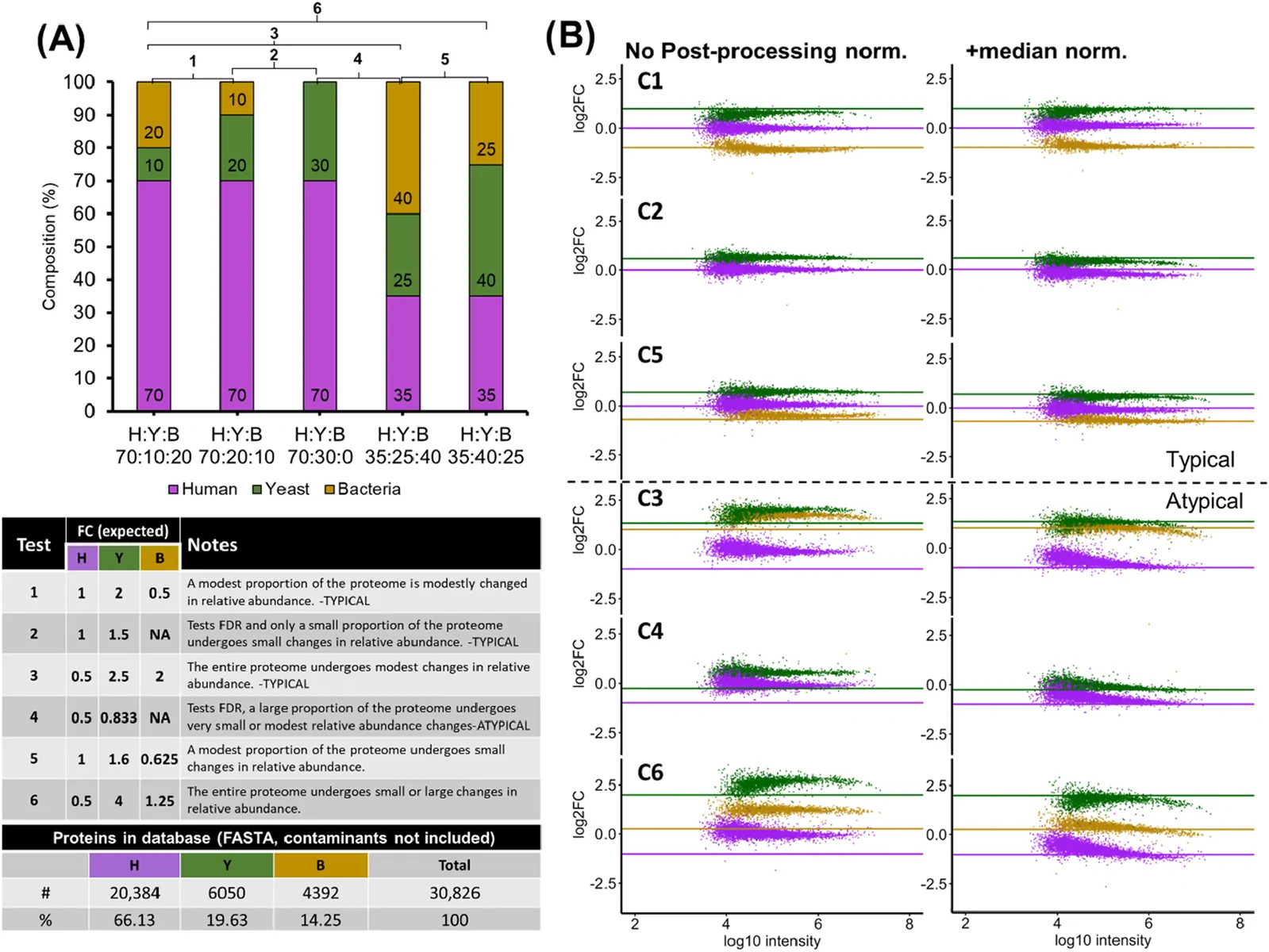
Three species CQEs. (A) Rationale for comparisons. (B) Median normalization alters distribution of measured protein group FC (points) around the ground truth (lines). FC shown here is the default postprocessing pipeline (2 peptides per protein, protein present in 3/5 replicates in both conditions, no other filtering).

We also considered the potential influence of LC–MS hardware on quantitative performance. Accordingly, the three-species CQEs were performed on a timsTOF HT using the optimal acquisition settings identified earlier (66 ms TART, 1000 ng load) and compared to a method routinely utilized in our lab on an Ultimate 3000 RSLC-Nano coupled to an Orbitrap Exploris 480. In addition to sample composition, instrumentation, and acquisition parameters, postprocessing decisions represent a critical factor in quantitative proteomics. Although numerous strategies exist for filtering and processing peptide-spectrum match (PSM) outputs, we initially focused on evaluating the effects of coefficient of variation (CV)-based filtering, exclusion of low-intensity precursors, minimum peptides-per-protein thresholds, and median normalization on protein group–level quantitative efficacy. For each comparison (C1–C6), 32 distinct postprocessing combinations were tested on both the timsTOF (Figure S3) and Orbitrap (Figure S6) data sets. Example outputs from three species CQEs following a postprocessing pipeline (1-peptide per protein minimum, median normalized, contaminants excluded) data sets from the timsToF-HT and E480 are provided (Tables S5–S6). The influence of postprocessing parameters on quantitative performance was broadly consistent between scenarios with typical versus atypical proteome changes, allowing a single representative example to suffice for most recommendations.

#### Recommendation: Median Normalization Is Usually Improving Data

Inspection of intensity and fold change profiles for the timsTOF HT (Figure 2B and Figure S3) and E480 (Figure S4 and S5) revealed that in typical comparisons (C1, C2, C5), fold-change distributions were already close to expected values without median normalization, whereas in atypical comparisons (C3, C4, C6) observed fold changes were substantially skewed upward. Median normalization exerted variable effects on quantitative efficacy across species and comparisons for both instrument platforms (Figures S3 and S5). In typical scenarios, modest decreases in quantitative performance were occasionally observed. For example, in C2 timsTOF data without additional processing (one peptide per protein), median normalization reduced yeast proteome accuracy from 97% to 94%, precision from 91% to 84%, and increased BS-FQR from 0% to 0.1%, while having negligible impact on human proteome accuracy and BS-FQR (precision could not be meaningfully assessed for the human proteome in this comparison).

In contrast, for atypical comparisons, median normalization consistently improved quantitative metrics across all species. In C3 timsTOF data, for example, median normalization increased human proteome accuracy from near 0% to ∼50%, precision from ∼20% to ∼90%, and reduced BS-FQR from ∼7% to ∼0%. For yeast and bacterial proteomes in the same comparison, accuracy rose from ∼10% and ∼2% to ∼95% and ∼96%, respectively, while precision remained at ∼100% and BS-FQR near 0%.

Results from the Orbitrap data set showed similar trends. In typical comparisons (C1, C2, C5), increasing the minimum peptide requirement generally improved quantitative metrics, with this effect being more pronounced when median normalization was not applied. Coefficient of variation (CV)-based filtering also enhanced quantitation in these cases, while median normalization had little effect. For atypical comparisons (C3, C4, C6), however, median normalization was critical. In C4, yeast protein precision dropped below 5% without normalization, while in C3, median normalization increased human protein precision from ∼85% to >98% and accuracy from <5% to substantially higher values. BS-FQR was also greatly reduced; in C3, human BS-FQR decreased from 1.5% to <0.1%, and in C4, absence of median normalization resulted in yeast BS-FQR values exceeding 90%. Interestingly, in C4, increasing the minimum peptide requirement slightly reduced yeast protein accuracy and worsened BS-FQR despite median normalization.

#### Recommendation: Exercise Caution in Quantitative Efficacy for Atypical Comparisons Where the Majority of the Proteome Changes

Atypical comparisons (C3, C4, and C6) exhibited substantial deviations from the expected ground-truth fold changes. Prior to applying quantitation metrics or filtering parameters, careful consideration of raw data processing is essential, particularly regarding the search strategy and search space. In these experiments, protein and precursor information were generated using the open-source software DIA-NN (version 1.9), which applies cross-run normalization by default.

For the initial analysis, all raw files, including both typical and atypical comparisons, were processed together in a single search. To assess the impact of search scope and cross-run normalization on quantitative metrics, separate searches were also performed for each comparison individually. Quantitative metrics for these approaches are shown in Figure 3. Overall, searching atypical experiments separately generally decreased quantitation performance, resulting in lower accuracy and higher BS-FQR values. This suggests that for experiments in which a large portion of the proteome is changing, a broader search space may be beneficial.

**Figure 3:**
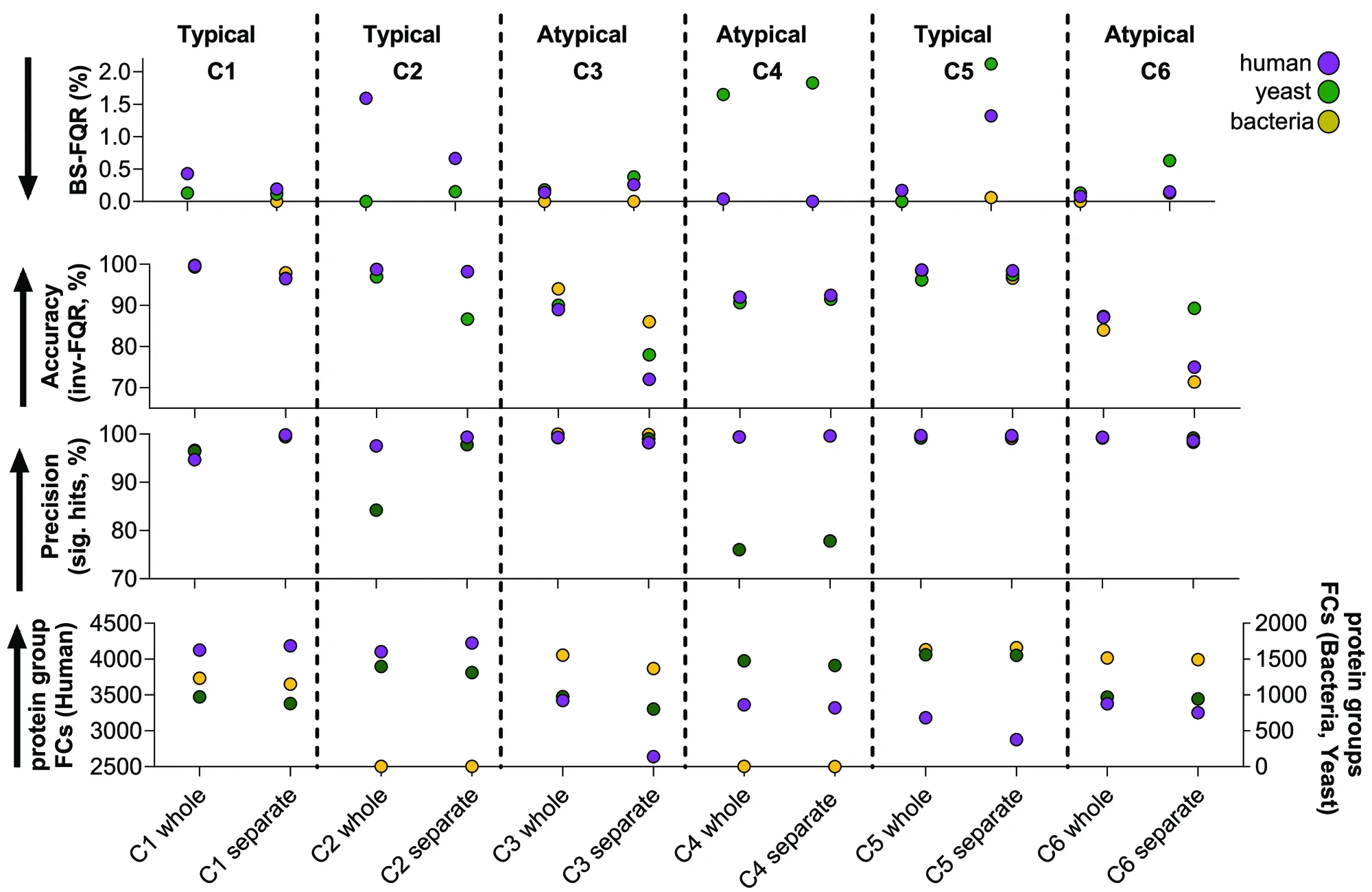
Search space has an impact on quantitative metrics. E480 Raw files were analyzed by DIA-NN separately for each condition (separate) or all at the same time (whole). Data shown underwent median normalization, protein present in 3/5 replicates in both conditions, with protein group CV filtering. Data are derived from 5 replicates.

For typical experiments, separate searches usually improved precision, but this came at the expense of accuracy, though BS-FQR values were improved for C1 and C2. These observations indicate that the effect of search space on quantitative metrics is experiment dependent. When atypical experiments were processed collectively, median normalization was critical for accurate quantitation, whereas it had minimal effect when experiments were searched individually. Thus, where median normalization may be inappropriate due to widespread proteome changes, independent searches without normalization may provide a more accurate approach. Figure 3 shows only median-normalized data for clarity.

#### Recommendation: Precursor Intensity Filtering Offers Minimal Quantitative Benefit

In order to test if low-intensity precursors affect the quantitation negatively, precursors were filtered by excluding the lowest 1% by mean intensity (calculated across all replicates within the comparison) for each comparison. To minimize the influence of extreme values, the upper and lower 1% of precursor intensities were excluded prior to analysis. Low-intensity precursors are more susceptible to poor identification and quantification, while very high-intensity precursors may disproportionately influence downstream analyses. As the intensity data were approximately normally distributed, this threshold corresponds to excluding values beyond approximately ±2.33 standard deviations from the mean. The 1% threshold is also consistent with commonly accepted filtering criteria within the proteomics community, where a 1% false discovery rate (FDR) is routinely applied to balance data quality and retention.

Across all comparisons and postprocessing conditions for both timsTOF and Orbitrap data sets (Figures S3 and S5), this filtering approach was occasionally mildly detrimental to identification rates and had negligible effects on quantitative efficacy metrics. In the typical comparison C1 (median normalization only, one peptide per protein, timsTOF data), the number of protein groups decreased slightly from 6695, 2315, and 1995 to 6695, 2311, and 1995 for human, yeast, and bacterial proteins, respectively. Corresponding accuracies declined marginally from 99.49%, 83.55%, and 98.17% to 98.49%, 83.49%, and 98.12%, while precision (for yeast and bacterial proteins) changed from 97.71% and 99.78% to 97.66% and 99.78%. BS-FQR values remained unchanged.

In the atypical comparison C6, protein group counts decreased minimally from 6165, 2319, and 2230 to 6164, 2319, and 2228 for human, yeast, and bacterial proteins, respectively. Accuracy values shifted from 59.90%, 81.93%, and 84.26% to 59.23%, 82.23%, and 83.89%, while precision changed from 93.20%, 99.66%, and 90.09% to 93.07%, 99.66%, and 90.53%. BS-FQR values remained constant with the exception of bacterial proteins, which showed a slight decrease from 0.058% to 0.054%. Similar patterns were observed in the Orbitrap data set, where precursor intensity filtering likewise had negligible effects on quantitative performance.

To test the impact of more extreme intensity-based filtering, the least intense 5, 10, 20, and 50% of precursors were excluded prior to protein group quantification. Protein group identifications and quantitative efficacy metrics were largely unaffected until the most extreme filtering was applied (Figure S6). In C1 exclusion of precursors with mean intensities below the percentiles <0, <1, <5, <10, < 20, and <50%. resulted in changes in numbers of proteins quantified of 6695, 6695, 6687, 6644, 6462, and 5216 for human, 2315, 2311, 2282, 2225, 2073, and 1414 for yeast, and 1995, 1995, 1989, 1975, 1921, and 1576 for bacteria. The benefits to quantitative efficacy were mostly positive (albeit usually minor) as filtering stringency increased, with some exceptions. In C1 for instance, yeast BS-FQR largely decreased between the <0–<50% filtering criteria; 0.26, 0.26, 0, 0, 0, 0.07, and in C5, bacterial BS-FQR increased; 0, 0, 0, 0.04, 0.04, and 0.10. In two typical comparisons where low ground truth fold changes were present, BS-FQR could be reduced from ∼1–2% to ∼0.1% by filtering the lowest 5% of precursors: Filtering precursors to exclude the lowest 5% reduced BS-FQR of yeast in C2 from 1.4 to 0.1% (expected FC 1.5-fold), and in C6 BS-FQR of bacteria (expected FC 1.25-fold) was reduced from 2.0 to 0.35%. Strangely, 5% filtering did not result in such drastic improvements in precision or accuracy in either case, indeed, accuracy was slightly worsened for bacteria in C6 (from 84 to 82%). Given this extensive testing for different precursor intensity filtering and its variable impacts (usually on proteins with low fold changes), we make the recommendation accordingly.

#### Recommendation: Low Protein Group Coefficient of Variation -or- a High Number of Peptides-Per Protein Are Both Strong Indicators of Quantitative Efficacy

Next we tested if proteins with high coefficient of variation (CV) or few quantified peptides were a source of inaccuracy. Protein groups were filtered CV, requiring a CV < 20% in each condition of a given comparison for inclusion. Additionally, proteins were filtered to include only those quantified by a minimum number of peptides, with thresholds ranging from one to four peptides per protein tested. While both CV- and peptide-number-based filtering predictably reduced total protein group identifications, substantial improvements in quantitative efficacy were observed across all comparisons for both timsTOF and Orbitrap data sets (Figure 4), with the exception of C4, which is discussed separately. Remarkably, although the absolute number of protein group identifications differs between the Orbitrap and timsTOF systems, trends in quantitative efficacy metrics were highly consistent, particularly for typical comparisons.

**Figure 4:**
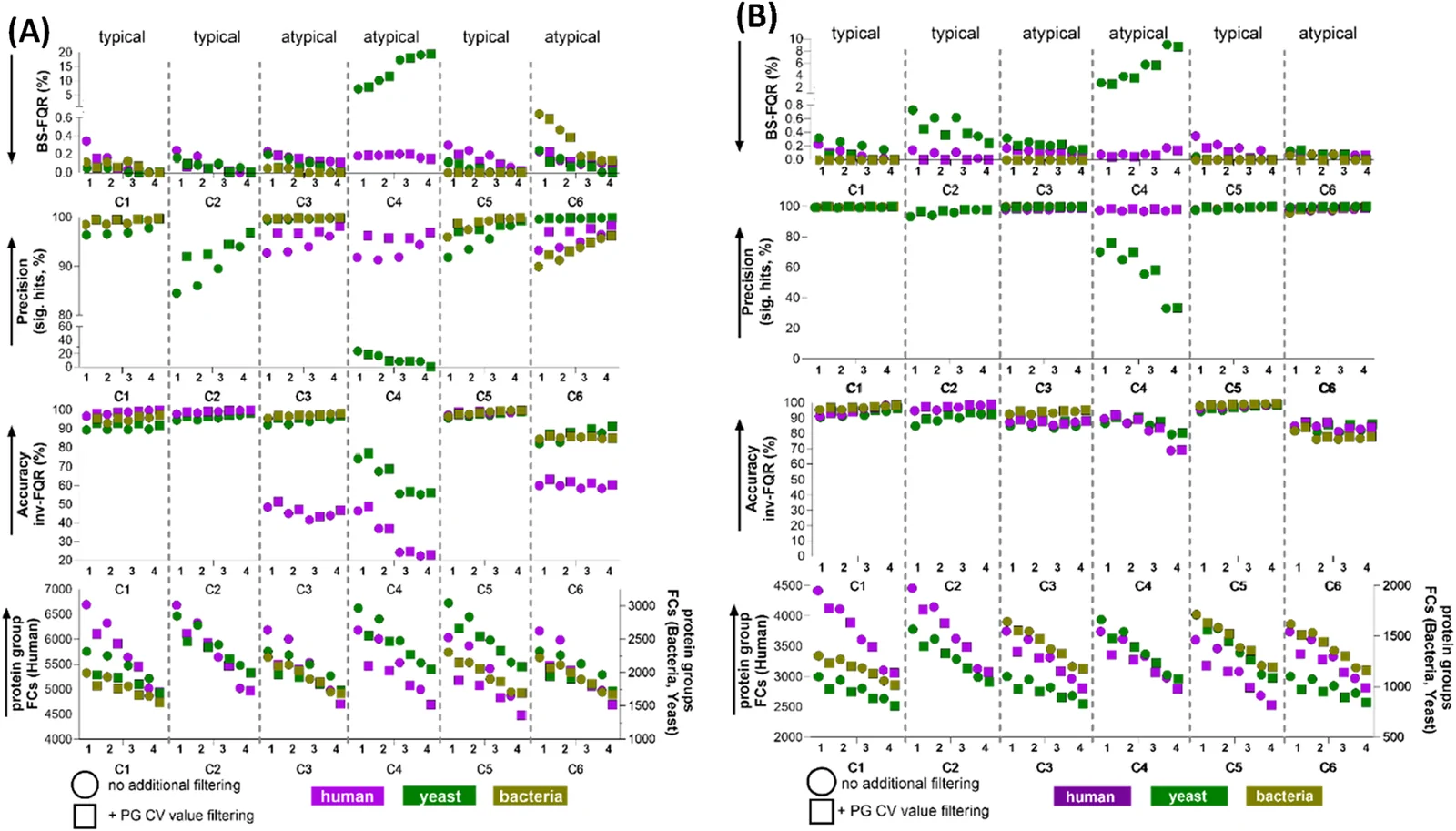
Filtering for peptide numbers per protein and protein CV have notable impacts on ID rates and quantitative efficacy in typical and atypical proteomics experiments. (A) timsTOF-HT (B) Orbitrap Exploris 480. Data shown underwent median normalization, protein present in 3/5 replicates in both conditions, ±protein group CV filtering (square and circle symbols) and variable filtering by minimum-number of peptides (numbers on *x*-axis). Data derived from 5 replicates.

In the typical comparison C1, applying a minimum threshold of four peptides per protein reduced human protein group counts by approximately 20% relative to one-peptide filtering (timsTOF: 6107 to 5018; Orbitrap: 4417 to 3413), yet quantitative efficacy improved substantially. For human proteins, accuracy increased from 96.6% to 99.7% on the timsTOF and from 91.1% to 98.2% on the Orbitrap, while BS-FQR decreased from 0.34% to 0% and from 0.22% to 0.02%, respectively. Similarly, in the atypical comparison C6, human protein group counts decreased by 10–20% with four-peptide filtering (timsTOF: 5479 to 4977; Orbitrap: 3740 to 2974), but quantitative performance improved: accuracy increased from 96.6% to 99.7% (timsTOF) and 91.1% to 98.2% (Orbitrap), precision increased from 93.2% to 95.4% and 97.9% to 98.8%, and BS-FQR decreased from 0.23% to 0.1% and 0.09% to 0.07%, respectively.

As expected, proteins quantified by higher numbers of peptides generally exhibited lower CV, and therefore the effect of CV filtering on quantitative efficacy was less pronounced for proteins with ≥3–4 peptides than for those with one or two peptides. For example, in the typical comparison C1, BS-FQR for human proteins in the timsTOF data set decreased substantially from 0.34% to 0.14% upon inclusion of CV filtering in the one-peptide data set, and from 0.22% to 0.10% for the Orbitrap. In contrast, when the same comparison was filtered for four peptides per protein, BS-FQR was already 0% for the timsTOF without CV filtering and decreased from 0.02% to 0% for the Orbitrap.

#### Recommendation: Single-Peptide Proteins Are Generally Well-Quantified and Rarely Cause Biologically Significant False Quantitation or Discovery

The data above showed that filtering out proteins with few quantified peptides, improved overall quantitation. Next we considered the performance of proteins quantified with a single peptide. We compared their precision, accuracy, and BS-FQR in each comparison (Figure 5), with and without protein group CV filtering in the timsTOF-HT data set.

**Figure 5:**
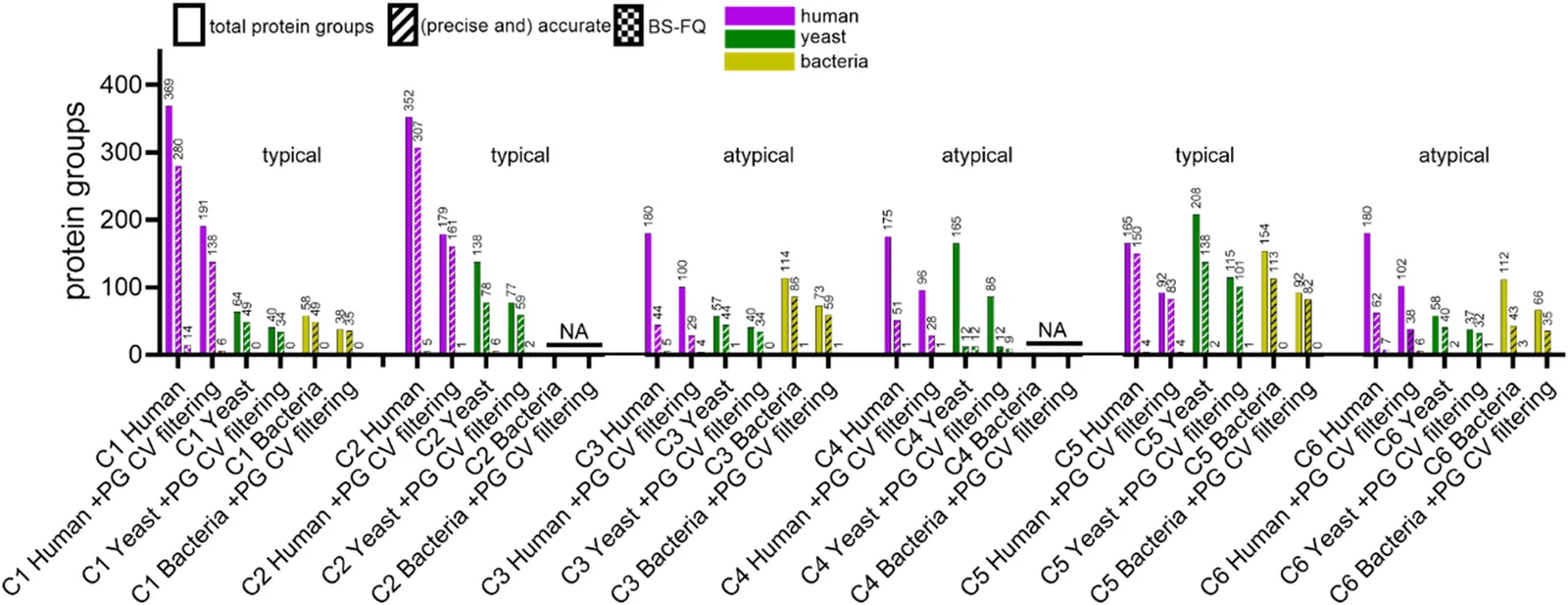
Single-peptide proteins and quantitative efficacy. Protein groups identified and quantified by a single peptide in the comparisons derived from the timsTOF-HT data set (median normalized, ± protein group CV filtering) were plotted for each comparison, alongside the total number, those that are accurate (and precise where appropriate), and those that contribute to BS-FQR. Data are derived from five replicates.

For typical comparisons (C1, C2, and C5), the majority of single-peptide protein fold changes are accurate, and very few represent biologically significant false quantitation. For example, in C5, of 165 single-peptide human proteins, 150 were accurate, and only 4 contributed to BS-FQR. For yeast and bacteria in the same comparison, 208 and 154 proteins were quantified, of which 138 and 113 were precise and accurate, and only 2 and 0 contributed to BS-FQR, respectively. While CV filtering modestly improved quantitative efficacy for single-peptide proteins, it did not fully eliminate false discoveries, and it also led to the loss of ∼50% of accurate human proteins in typical comparisons (C1, C2, C5) and ∼20% of accurate and precise proteins in atypical comparisons across human, yeast, and bacterial data sets.

Regarding false discoveries, a small number of *E. coli* proteins (≤10) were detected in the HYB 70:30:0 condition, where they should not have been present, leading to some *E. coli* protein groups in comparisons C2 and C4 (Figure S7 and Table S7). Applying an additional filter to include only proteotypic precursors—precursors unique to a single protein—had minimal impact on these falsely discovered *E. coli* proteins. Notably, all of these proteins were quantified by 4–39 peptides across the comparison, indicating that removing single-peptide hits does not prevent these apparent false discoveries. Protein group CV filtering, however, removed most falsely quantified proteins, which were generally close to no-fold change (within ±1.5-fold) or changed in the expected direction for the comparison (Figure S7B). In comparison C4, one *E. coli* protein remained significantly decreased beyond the ±0.5-FC cutoff (−2.26-fold change), regardless of CV filtering.

#### Recommendation: Apply a Minimum Fold-Change Threshold of ±1.5 to Limit False Discoveries in DIA-LFQ

To prevent proteins with low quantitative accuracy being annotated as “significant”, a fold-change cutoff is often applied alongside appropriate statistical tests. Here, a ±1.5-FC threshold (0.75–1.5-fold) was used, although more stringent cutoffs could be applied if desired. In typical comparisons where the human proteome is expected to be unchanged, small fold changes were still observed for all quantified proteins, generally close to 1 (i.e., no true fold change; Figure 2B).

These minor, artifactual abundance shifts were measured consistently enough that a substantial fraction of proteins appeared statistically significant, despite stringent Benjamini-Hochberg adjustment of *p*-values. In C1 and C2 comparisons, for example, where human proteins stayed constant, over 50% of human proteins were classed significant by p-value alone (Figure S8). By applying the ±1.5-FC cutoff, inference of “biological significance” for these proteins is appropriately restricted, resulting in very low BS-FQR for human proteins (Figure 4, Figures S3 and S5). Implementing this fold-change threshold also effectively mitigates the false quantification and discovery of proteins described in the previous section (Figure S7). Should an experimenter require smaller thresholds for significance, it might be desirable to reassess quantitative efficacy using a CQE with smaller ground-truth fold changes, and assigning a target (i.e., lower) ± FC.

#### Recommendation: Exclusion of Precursors with Low Points Per Peak May Not Benefit Protein Group Quantitation, Though Effects Are Instrument- and Method-Dependent

A contentious parameter in untargeted proteomics is the minimum number of points per peak (PPP) required for acceptable quantitative efficacy, and whether protein group quantitation should rely on precursors with low PPP. To assess this, different PPP filters were applied to timsTOF and Orbitrap data sets. For each precursor, DIA-NN reports the full width at half-maximum (FWHM) in minutes, allowing calculation of approximate PPP-FWHM based on the MS/MS cycle time (1.2 s for timsTOF and 1.6 s for Orbitrap). A range of PPP filtering parameters was tested (Figure 6).

**Figure 6:**
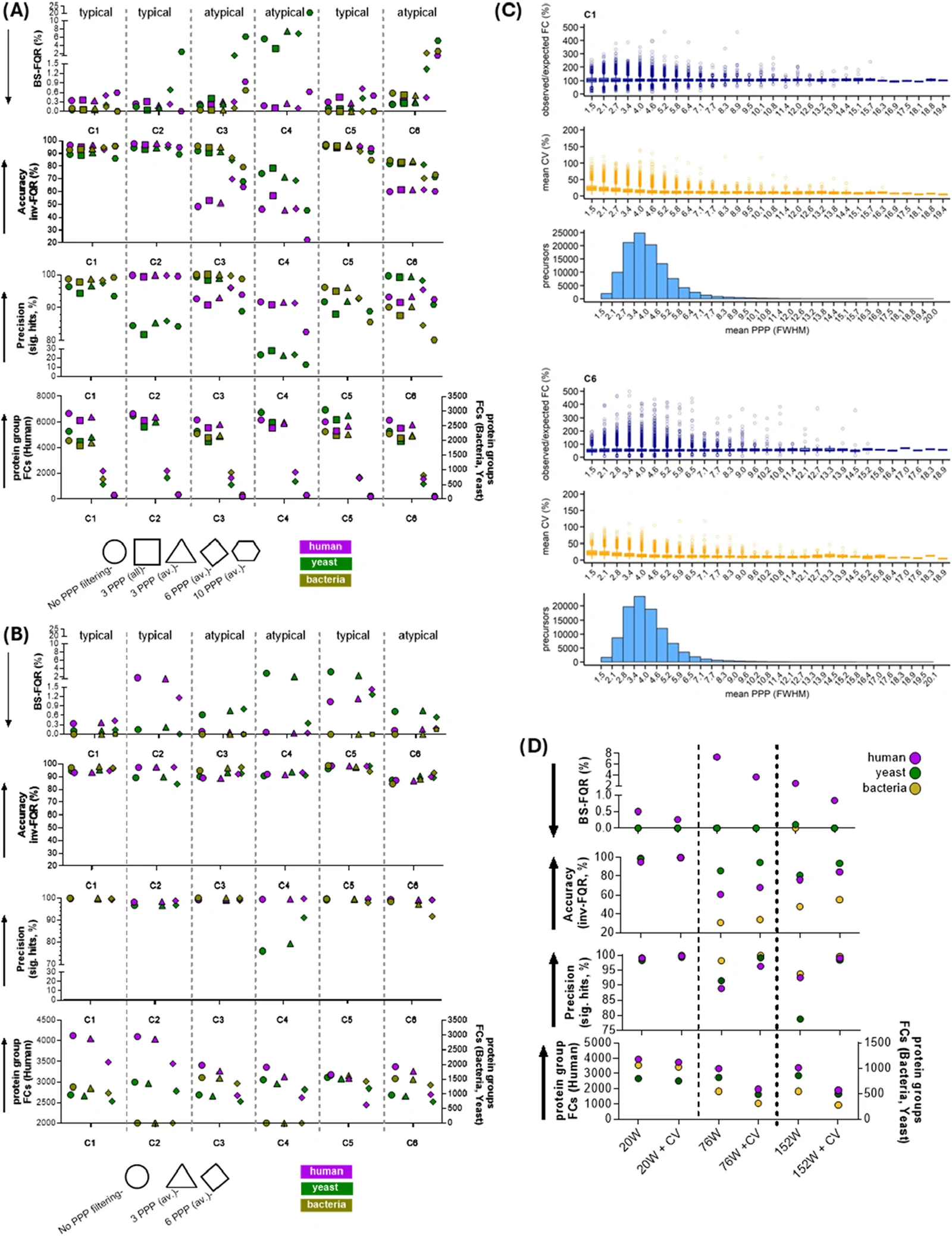
The impact of Precursor PPP filtering on Quantitative Efficacy. (A, B) timsTOF and Orbitrap-quantified protein groups subjected to different modes of PPP-filtering. (all) = global precursor filtering, (av.) = precursor filtering based on mean PPP-FWHM across all replicates from all conditions in a given comparison. (C) timsTOF precursors (with no filtering) were placed into 30 bins based on their PPP, for each comparison. For each precursor bin, the number of precursors was plotted-lowest histogram, as was the median CV (%) and deviation from ground truth (observed/expected fold change), (%)-middle and upper boxplot. Boxplots show median, quartiles, outliers. Data are derived from 5 replicates. (D) Quantified protein groups using MS data acquired with different DIA window schemes on the E480 (20, 76, and 152 windows), both with and without CV filtering.

For timsTOF data (Figure 6A), a global filter of 3 PPP-FWHM applied to all precursors before assembling protein groups and performing single-peptide-per-protein analysis unexpectedly had a mildly detrimental effect on most quantitative metrics across all species. In C1, human, yeast, and bacterial protein group fold changes decreased from 6695/2315/1995 to 6143/1962/1825, yeast/bacterial precision dropped from 96.4%/98.6% to 94.4%/97.7%, accuracy shifted from 96.6%/89.3%/92.6% to 95.1%/88.6%/92.9%, and BS-FQR slightly increased. The bacterial condition showed a minimal improvement (≤0.5%), but most metrics worsened. Missing value inspection revealed that filtering disproportionately removed precursors even within replicates of the same condition.

Filtering by *mean* PPP-FWHM across replicates (PPP­(av.)), separately for each comparison, produced similar metrics to no filtering, though with slightly reduced protein group IDs (C1 FCs dropped from 6695/2315/1995 to 6411/2114/1915). Moderate PPP thresholds (3 PPP­(av.)) had minimal effect, but stricter cutoffs (6 or 10 PPP­(av.)) caused severe ID losses and worsened quantitative efficacy—especially in atypical comparisons—while increasing BS-FQR (e.g., C6 bacterial BS-FQR rose from 0.58% to 2.24% at 6 PPP­(av.)). In the most stringent case, IDs dropped below 400 (human) and 200 (yeast/bacteria) for all comparisons.

Precursor PPP distribution analysis (Figure 6C and Figure S9) showed a modal average of 3.4–4.0 PPP-FWHM, with median precursor CVs ∼25% in most bins (rising to ∼50% at 1.5–2.1 PPP-FWHM). Despite this, observed/expected protein group fold changes were ∼100% across bins, suggesting that protein-level quantitation is largely independent of individual precursor PPP in the timsTOF data sets.

By contrast, Orbitrap E480 data benefited more from PPP filtering (Figure 6B). A 3 PPP-FWHM threshold reduced IDs only slightly (3–4%), indicating most precursors already exceeded ∼8 PPP. Increasing to 6 PPP-FWHM caused larger ID losses (19–23%) but generally improved precision, accuracy, and BS-FQR (e.g., C4 yeast precision rose from 74% to 90%). Some exceptions occurred (e.g., C6 yeast precision dropped from 98% to 90%), indicating instrument- and experiment-specific effects.

The relatively limited impact of PPP filtering in timsTOF data can likely be attributed to previous careful DIA method optimization, balancing PPP, cycle time, and reproducibility to yield consistently low CVs. To test the deliberate changes in PPP, we analyzed C1 samples with three DIA schemes on the Exploris 480:

- **152 DIA windows** with 2 *m*/*z* overlap (cycle time 12.6 s, ∼3 PPP-FWHM)
- **76 DIA windows** with no overlap (cycle time 6.3 s, ∼4.5 PPP-FWHM)
- **20 DIA windows** with 12 *m*/*z* overlap (cycle time 1.6 s, ∼5.9 PPP-FWHM)

Quantitative efficacy metrics (Figure 6D) show that the 20-window scheme delivered the strongest performance across all measures. Methods with fewer PPP (e.g., 152-window) suffered greater ID losses upon CV filtering, likely due to higher precursor/protein detection variability. Comparing the overlapping 152-window to the nonoverlapping 76-window scheme suggested that window overlap can modestly improve accuracy and BS-FQR when combined with CV filtering.

Overall, PPP filtering effects are both instrument- and method-dependent: there was minimal benefit (and some detriment) for the timsTOF when acquisition was already optimized, but potential improvements for Orbitrap data sets at moderate PPP thresholds.

#### Recommendation: Not Only Identification Rates, but Also Quantitative Efficacy Must Be Considered for Low-Input or Higher-Throughput Proteomics Approaches

While most current proteomics experiments utilize hundreds of nanograms to micrograms of material at modest or low throughput, the field is trending toward shorter run times per sample and smaller amounts of input material. Work by Gatto et al.^23^ has shown The accuracy and reproducibility of quantitative results are influenced by multiple factors spanning experimental design, sample preparation, data acquisition, and downstream data analysis, In parallel, new hardware^8,24^ and novel or adapted acquisition modes are being reported. Most studies emphasize protein identification rates, but quantitative efficacy must also be evaluated before widespread adoption of these methods, and any limitations must be clearly defined.

We applied this principle when testing high-sensitivity methods on the timsTOF-HT. Initially, 250 pg of tryptic HeLa digest was analyzed by diaPASEF using 4, 6, or 16 frames, with TARTs of 100, 200, 300, or 400 ms, and either standard (*m*/*z* 300–1200) or focused (*m*/*z* 450–950) mass ranges, at a throughput of 40 SPD (Figure S10). Overall, 200–300 ms TART, 4W methods were most optimal based on observed metrics (protein group IDs, protein group CV, and mean precursor PPP), achieving ∼3000 protein groups, protein group CV < 15%, and ∼2.5–3 mean PPP-FWHM. While these metrics are good given that the hardware is not optimized for single-cell experiments, quantitative efficacy cannot be inferred from these metrics alone.

Therefore, a single CQE was performed using 250 pg of the defined species tryptic digest (C1, H:Y:B 70/20/10 → 70/10/20), testing key postprocessing parameters (Figure 7): Minimum 1- or 4-peptides-per-protein filtering, with or without protein group CV filtering, and re-evaluating precursor intensity and PPP filtering under these high-sensitivity conditions. Across all processing conditions, log2 fold-change distributions suggested compression, particularly for bacterial proteins, with observed fold changes skewed toward zero (Y; B log2FC medians ∼0.75; −0.55). This was reflected in quantitative metrics: with minimal filtering, bacterial precision was ∼40% and accuracy ∼30%, while yeast and human performed better (∼70% precision, ∼75% accuracy). BS-FQR remained low across species (H;Y;B = 1%;0%;0%).

**Figure 7:**
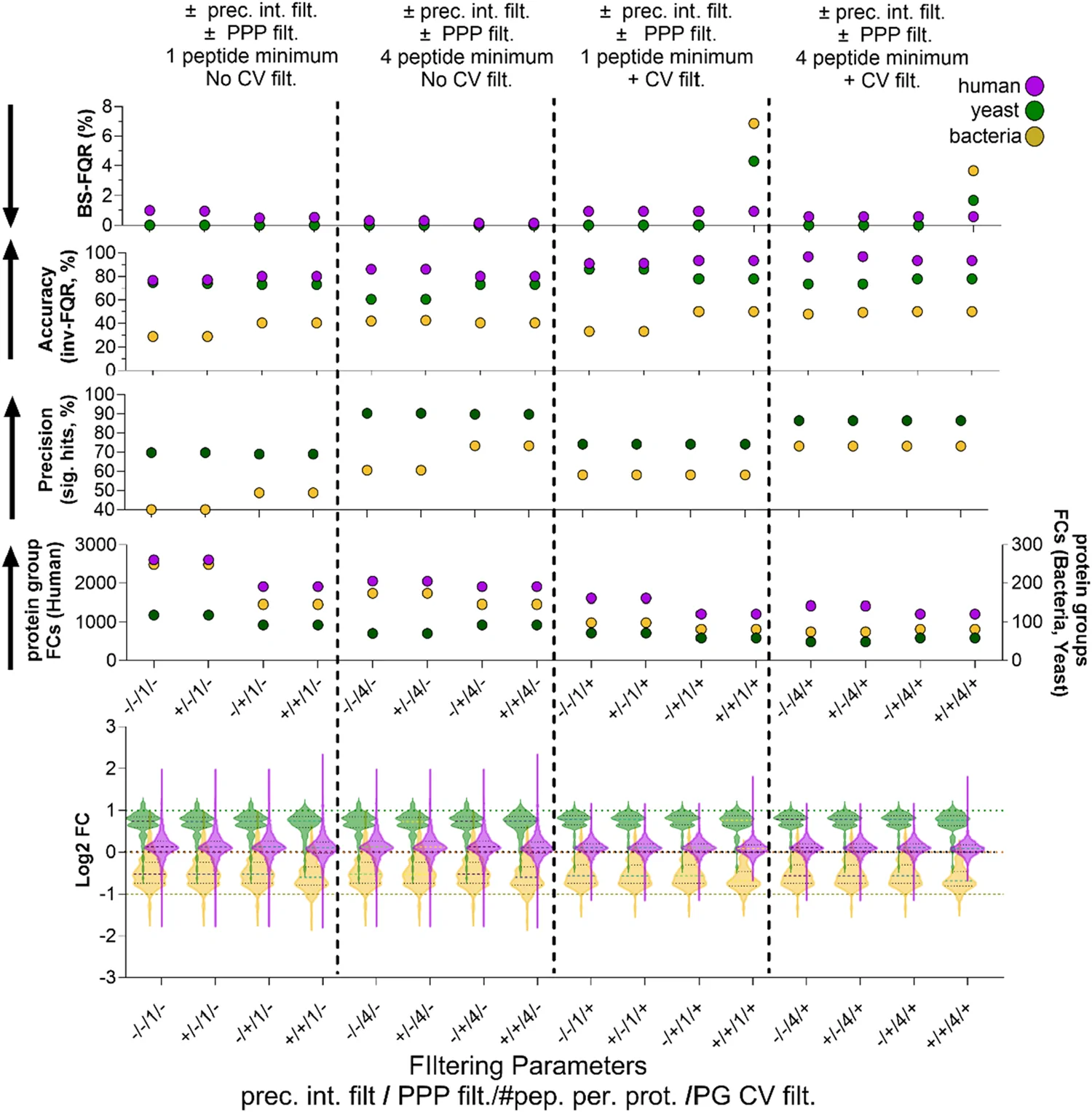
Quantiative efficacy of the timsTOF-HT at single-cell level sensitivity. Data underwent median normalization, protein present in ≥3/5 replicates in both conditions. Then as indicated, ± precursor intensity filtering (bottom 1% excluded), ± PPP-filtering (mean PPP-FWHM ≥ 3 across all replicates from all conditions in a given comparison), minimum-number of peptides, and ± protein group CV filtering (square and circle symbols). Data derived from 5 replicates.

Filtering by ≥4-peptides-per-protein generally improved quantitative efficacy. Compared with ≥1-peptide-per-protein filtering, yeast and bacterial precision increased from 69.8%;40.1% to 90%;61%, H;Y;B accuracy from 76.6%;74.8%;28.9% to 86.0%;60.6%;42%, and BS-FQR decreased slightly from 1%;0%;0% to 0.3%;0%;0%. Protein group CV filtering also improved quantitative metrics, while precursor intensity filtering had minimal impact. Points-per-peak (PPP) filtering provided moderate benefit, improving precision (Y;B 69.8%;40.1% → 69.0%;48.9%) and accuracy (H;Y;B 76.6%;74.8%;28.9% → 80.1%;73.1%;40.4%), with minor effects on BS-FQR. Interestingly, applying all high-stringency filters together (PPP, intensity, and protein group CV) increased BS-FQR for yeast and bacterial proteomes, indicating overly stringent filtering can be counterproductive.

Overall, the CQE revealed that this diaPASEF scheme on a timsTOF-HT for single-cell and ultrahigh-sensitivity proteomics exhibits fold-change compression, reducing quantitative accuracy for proteins with differential abundance. Nevertheless, the low BS-FQR (<1%) indicates that proteins identified as significantly changing (±1.5-FC) are likely true positives, especially when quantified by ≥4 peptides and/or precursors ≥3 average PPP. Importantly, ≥4-peptides-per-protein filtering alone enhanced quantitative efficacy more effectively than ≥1-peptide-per-protein filtering combined with PPP filtering. When PPP filtering is applied, high peptide-per-protein filtering should also be used to maximize quantitative efficacy without further loss of protein fold-change. For example, moving from ≥1-peptide-per-protein + PPP filtering to ≥4-peptides-per-protein + PPP filtering increased Y;B precision from 69%;40.2% to 89.8%;73.3%, maintained H;Y;B accuracy at 80.1%;73.1%;40.4%, and improved BS-FQR from 0.5%;0%;0% to 0.14%;0%;0%, while protein group fold-changes remained constant (1915/93/146).

## Discussion

The widespread adoption of data-independent acquisition (DIA) on modern mass spectrometers has dramatically improved throughput and sensitivity, enabling experiments such as single-cell proteomics that seemed unattainable only a few years ago. While much of the field has focused on maximizing the number of identified proteins, less attention has been paid to the quantitative efficacy of these approaches.

Controlled quantitative experiments (CQEs) have proven invaluable for benchmarking emerging instruments and methods.^13,18,19,21,25^ In this study, we employed a range of CQEs to systematically evaluate acquisition parameters and data-processing strategies, and we present a set of practical recommendations for the community.

First, we advocate the use of simple two- or three-species CQEs to optimize any novel DIA (or DDA) workflow. This design provides a robust framework for balancing protein identifications with quantitative accuracy, and it is particularly informative across different sample loads, where fill times may need adjustment. As expected, lower sample inputs reduced protein identifications and compromised quantitation. Nevertheless, even at 250 pg loading, overall proteome-level quantitation remained robust, although with compressed ratios and decreased precision compared to standard protein inputs. These observations are directly relevant to the rapidly expanding field of single-cell proteomics, where such trade-offs are unavoidable and must be carefully considered.

For most conventional proteomics applications—where only a small subset of proteins changes abundance—standard workflows already perform well. However, atypical experiments involving large-scale proteome changes may require tailored approaches. Our data suggest that normalization, while generally beneficial, can in some cases introduce distortions, particularly when combining data sets from typical and atypical experiments.

CQEs also enable researchers to assess which instrument platform is most suitable for a given application. By comparing two widely used instruments, we identified performance differences that highlight complementary strengths and weaknesses. For instance, the rapid scan speed of TOF analysers appears to mitigate quantitation issues associated with reduced points per peak, in contrast to slower Orbitrap analysers. Conversely, we observed ratio compression for high-abundance precursors on the timsTOF, potentially arising from space-charge effects or detector saturation. Importantly, this effect had minimal impact at the protein level, as high-abundance proteins were typically quantified by multiple peptides.

In addition, CQEs are a powerful tool for evaluating false discovery rates (FDR) of search algorithms. Across our analyses, we detected very few false-positive protein identifications. In one stringent comparison (C4), where no *E. coli* should have been detected in one condition, only a small number of *E. coli* proteins were identified, well below the nominal 1% FDR threshold. This suggests that current tools may be even more stringent than their stated FDR estimates.

Overall, our results reinforce that established proteomics workflows already deliver excellent quantitation, low false quantitation rates (FQR), and robust FDR control. Even proteins supported by a single peptide were generally well quantified, underscoring the reliability of modern DIA-based proteomics. These findings should reassure the community that proteomics is a powerful and trustworthy quantitative approach, capable of driving biological and medical discoveries. This is particularly timely as non-mass spectrometry-based platforms are being marketed as high throughput proteomics solutions, often without equivalent benchmarking against established methods.

Finally, we summarize our recommendations in Table 2, which outline best practices for acquisition, processing, and evaluation of DIA data. We hope these guidelines will assist researchers in designing, executing, and interpreting proteomics experiments, and will promote robust and reproducible applications of DIA across the field.

**Table 2:** Recommendations for Quantitative Proteomics using DIA-LFQ

(1) *Recommendation: Low protein group coefficient of variation -or- a high number of peptides-per protein are both strong indicators of quantitative efficacy*.
(2) *Apply a Minimum Fold-Change Threshold of ±1*.*5 to Limit False Discoveries in DIA-LFQ*.
(3) *Not only identification rates*, *but also quantitative efficacy must be considered for low-input or higher-throughput proteomics approaches*.
(4) *Single-peptide proteins are generally well-quantified and rarely cause biologically significant false quantitation or discovery*.
(5) *Exclusion of precursors with low points per peak may not benefit protein group quantitation*, *though effects are instrument- and method-dependent*.
(6) *Precursor Intensity Filtering offers Minimal Quantitative Benefit*.
(7) *Median normalization is usually improving data*.
(8) *Perform Two-Species CQEs for initial DIA parameter optimization*.
(9) *Exercise caution when considering quantitative efficacy for atypical comparisons*, *where the majority of the proteome changes*.

## Supplementary Material

















## Data Availability

The mass spectrometry proteomics data have been deposited to the ProteomeXchange Consortium via the PRIDE^13^ partner repository with the data set identifier PXD068065 for the E480, and PXD068192 for the timsTOF-HT.

## Notes

Summary quality control metrics for all load and TART conditions (Figure S1); normalization skews data away from the ground truth when the majority of the proteome is expected to change in abundance (Figure S2); post processing impacts quantitative efficacy in typical and atypical proteome comparisons derived from timsToF-HT analyses (Figure S3); median normalization alters distribution of measured protein group FC for E480 data (Figure S4); post processing impacts quantitative efficacy in typical and atypical proteome comparisons derived from Orbitrap Exploris 480 analyses (Figure S5); precursor intensity filtering impacts quantitative efficacy in typical and atypical proteome comparisons derived from timsToF-HT analyses (Figure S6); falsely discovered changes in relative abundance are minimal, but may be present in typical proteomics analyses (Figure S7); human proteins and those with statistical significance in comparisons with no ground-truth abundance change (Figure S8); relationship between precursor PPP (fwhm), precursor CV, and precursor deviation from ground truth (Figure S9); high sensitivity diaPASEF optimization-ID rates and reproducibility for single-cell level loads on the timsToF HT (Figure S10) (PDF)
E480_acquisition_schemes (Table S1) (XLSX)
HT_acquisition_scheme_16W2F (Table S2) (XLSX)
HT_SCP_acquisition_scheme_4W2F (Table S3) (XLSX)
HT_merged_output_2Species (Table S4) (XLSX)
480_example_output (Table S5) (XLSX)
HT_example_output (Table S6) (XLSX)
E_coli_Protein_False_Discorvery_C2_C4 (Table S7) (XLSX)

## Abbreviations

AGC: Automatic gain control
BS-FQR: Biologically significant false quantitation rate
CQE: Controlled quantitative experiment
CV: Coefficient of variation
DDA: Data dependent acquisition
DIA: Data independent acquisition
FDR: False discovery rate
FQR: False quantitation rate
FWHM: Full width half-maximum
IAA: Iodoacetamide
ID: Identification
LC: Liquid chromatography
LC-MS/MS: Liquid chromatography mass spectrometry
LFQ: Label free quantification
MS: Mass spectrometry
PSM: Peptide spectrum match
QC: Quality control
SDS: Sodium dodecyl-sulfate
TART: TIMS accumulation and ramp time
TCEP: Tris­(2-carboxyethyl)­phosphine hydrochloride
TDC: Target decoy competition
TEAB: Triethylammonium bicarbonate buffer
TIMS: Trapped ion mobility spectrometry
TOF: Time of flight
