# Supplementary material for: Recommendations for Quantitative Data-Independent Acquisition (DIA) Proteomics Using Controlled Quantitative Experiments (CQEs)
Source: J Proteome Res. 2026 Oct 22;25(11):5817–30. doi: 10.1021/acs.jproteome.6c00328 (PMC13750105; doi:10.1021/acs.jproteome.6c00328)
Supplement: Supplementary file 1 [file pr6c00328_si_001.pdf]

Recommendations for Quantitative Data-Independent Acquisition (DIA) Proteomics using Controlled Quantitative Experiments (CQEs)

Andrew M Frey[1,2#], Frances Sidgwick[1#], Andrew Porter[1], Pawel Palmowski[1], Matthias Trost[1,3*]

[1]Biosciences Institute, Newcastle University, Newcastle-upon-Tyne, NE2 4HH UK
[2]Translational and Clinical Research Institute, Newcastle University, Newcastle-upon-Tyne, NE2 4HH, UK
[3] Division of Molecular & Cellular Function, University of Manchester, Manchester, M13 9PT, UK

[#]:these authors contributed equally

*:Correspondence to: matthias.trost@manchester.ac.uk

Table of Contents

Figure S1. Summary quality control metrics for all load and TART conditions. S2
Figure S2. Normalisation skews data away from the ground truth when the majority of the proteome is expected to change in abundance. S3
Figure S3. Post processing impacts quantitative efficacy in typical and atypical proteome comparisons derived from timsToF-HT analyses. S3
Figure S4. Median normalisation alters distribution of measured protein group FC for E480 data. S10
Figure S5. Post processing impacts quantitative efficacy in typical and atypical proteome comparisons derived from Orbitrap Exploris 480 analyses. S16
Figure S6. Precursor intensity filtering impacts quantitative efficacy in typical and atypical proteome comparisons derived from timsToF-HT analyses. S17
Figure S7. Falsely-discovered changes in relative abundance are minimal, but may be present in typical proteomics analyses. S18
Figure S8. Human proteins and those with statistical significance in comparisons with no ground-truth abundance change. S19
Figure S9. Relationship between precursor PPP (FWHM), precursor CV, and precursor deviation from ground truth. S20
Figure S10. High sensitivity diaPASEF optimisation-ID rates and reproducibility for single-cell level loads on the timsToF HT. S21
Table S1. E480_acquisition_schemes.xlsx
Table S2. HT_acquisition_scheme_16W2F.xlsx
Table S3. HT_SCP_acquisition_scheme_4W2F.xlsx
Table S4. HT_merged_output_2Species.xlsx
Table S5. 480_example_output.xlsx
Table S6. HT_example_output.xlsx
Table S7. E_coli_Protein_False_Discorvery_C2_C4.xlsx

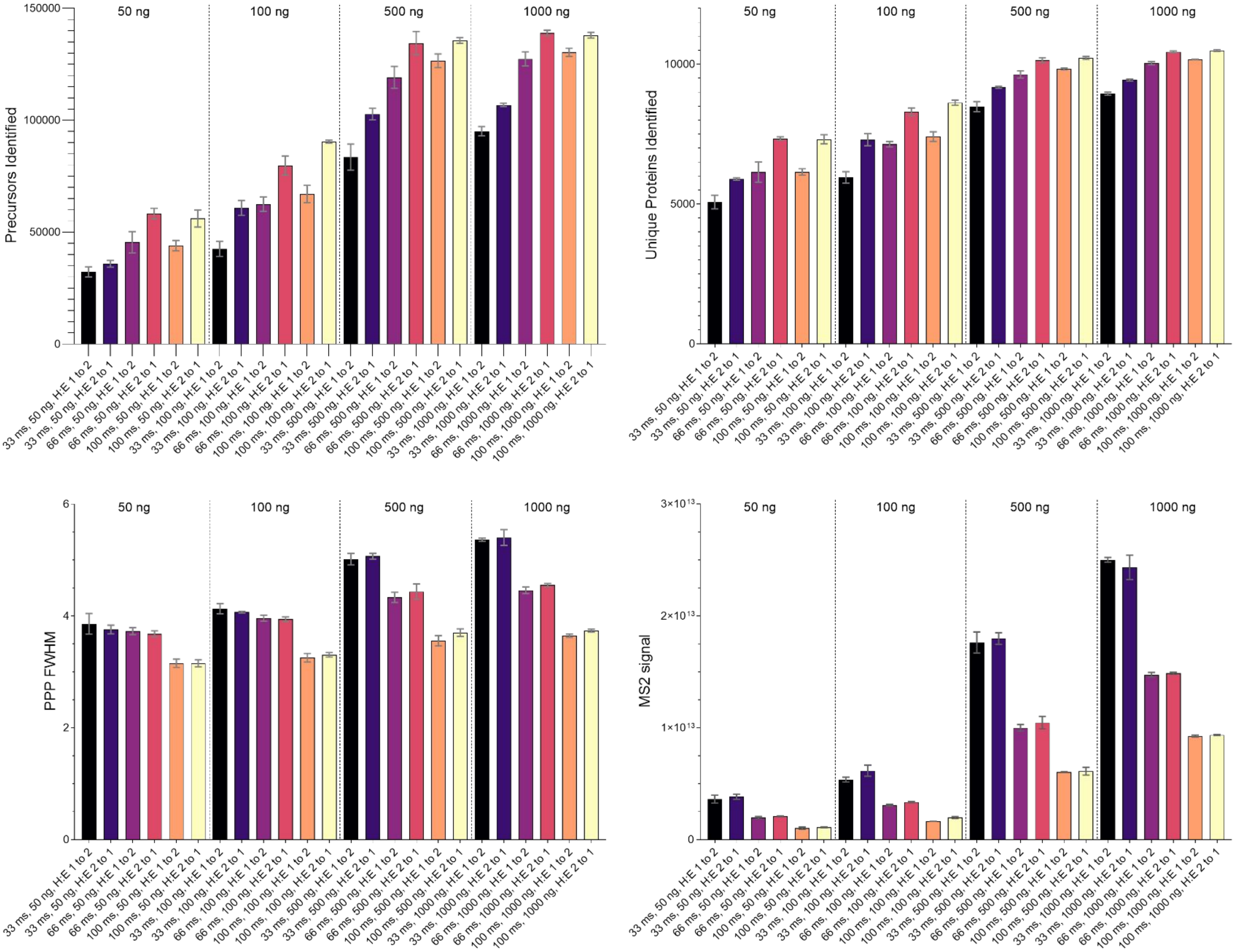

Figure S1. Summary quality control metrics for all load and TART conditions. Data shown are the mean of three replicates, error bars = standard deviation. PPP FWHM = points per peak, full width at half maximum.

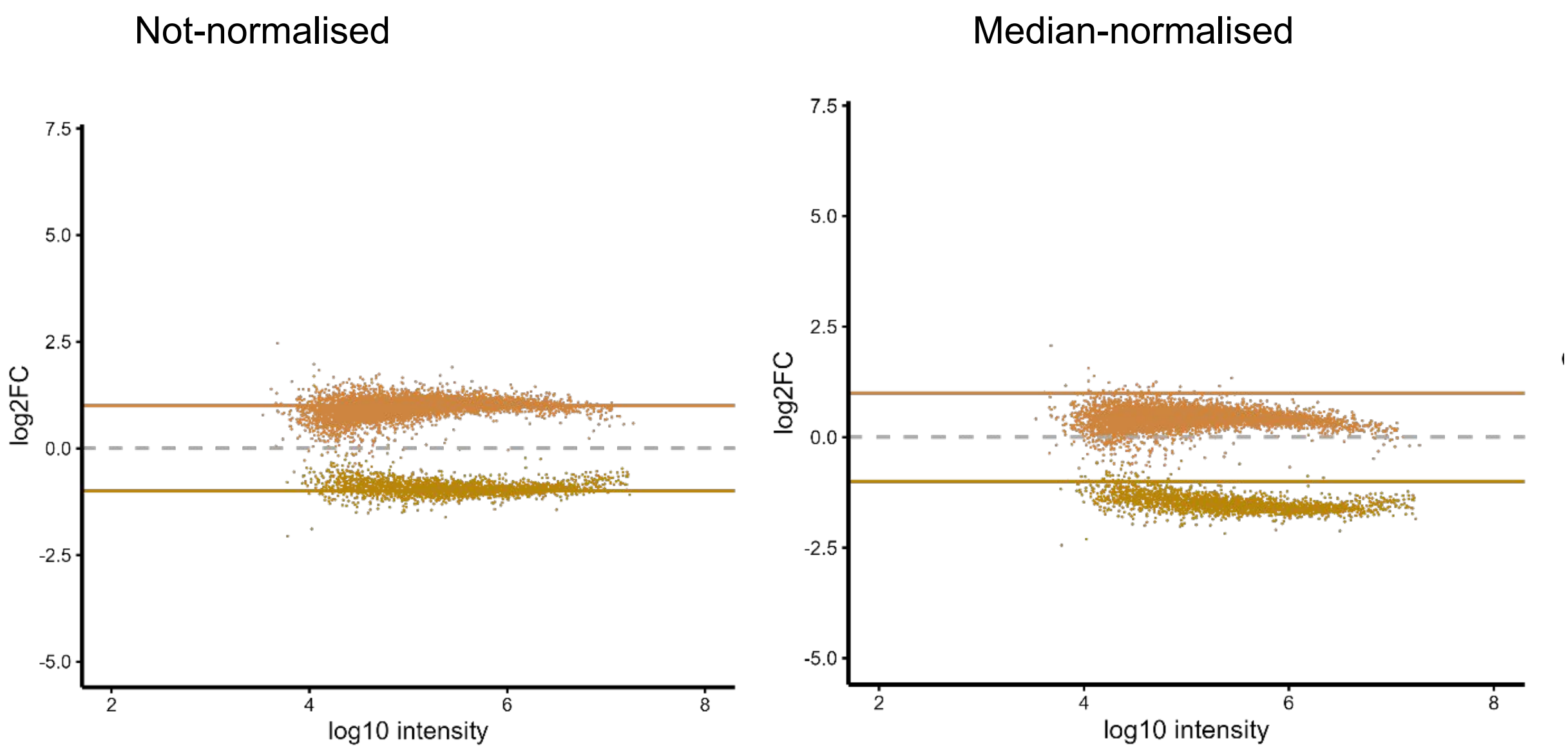

Figure S2. Normalisation skews data away from the ground truth when the majority of the proteome is expected to change in abundance. Distribution of (log10) protein group intensities against (log2) fold change for all conditions. Terracotta = Human, Khaki = Bacteria. Data shown are derived from three replicates.

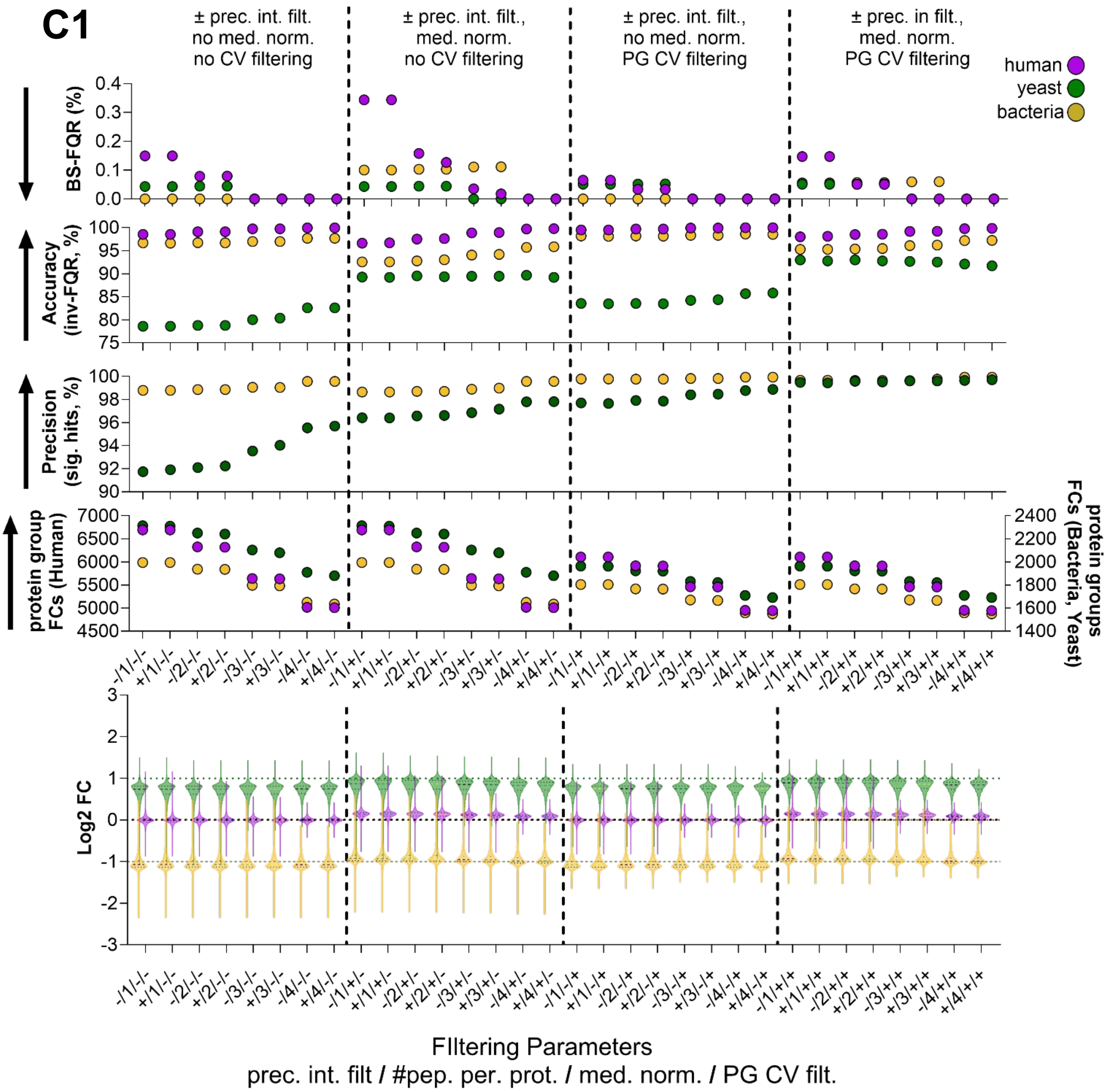
C1
± prec. int. filt. no med. norm. no CV filtering
± prec. int. filt., med. norm. no CV filtering
± prec. int. filt., no med. norm. PG CV filtering
± prec. in filt., med. norm. PG CV filtering
human
yeast
bacteria
BS-FQR (%)
Accuracy (inv-FQR, %)
Precision (sig. hits, %)
protein group FCs (Human)
protein groups FCs (Bacteria, Yeast)
Log2 FC
FIltering Parameters
prec. int. filt / #pep. per. prot. / med. norm. / PG CV filt.

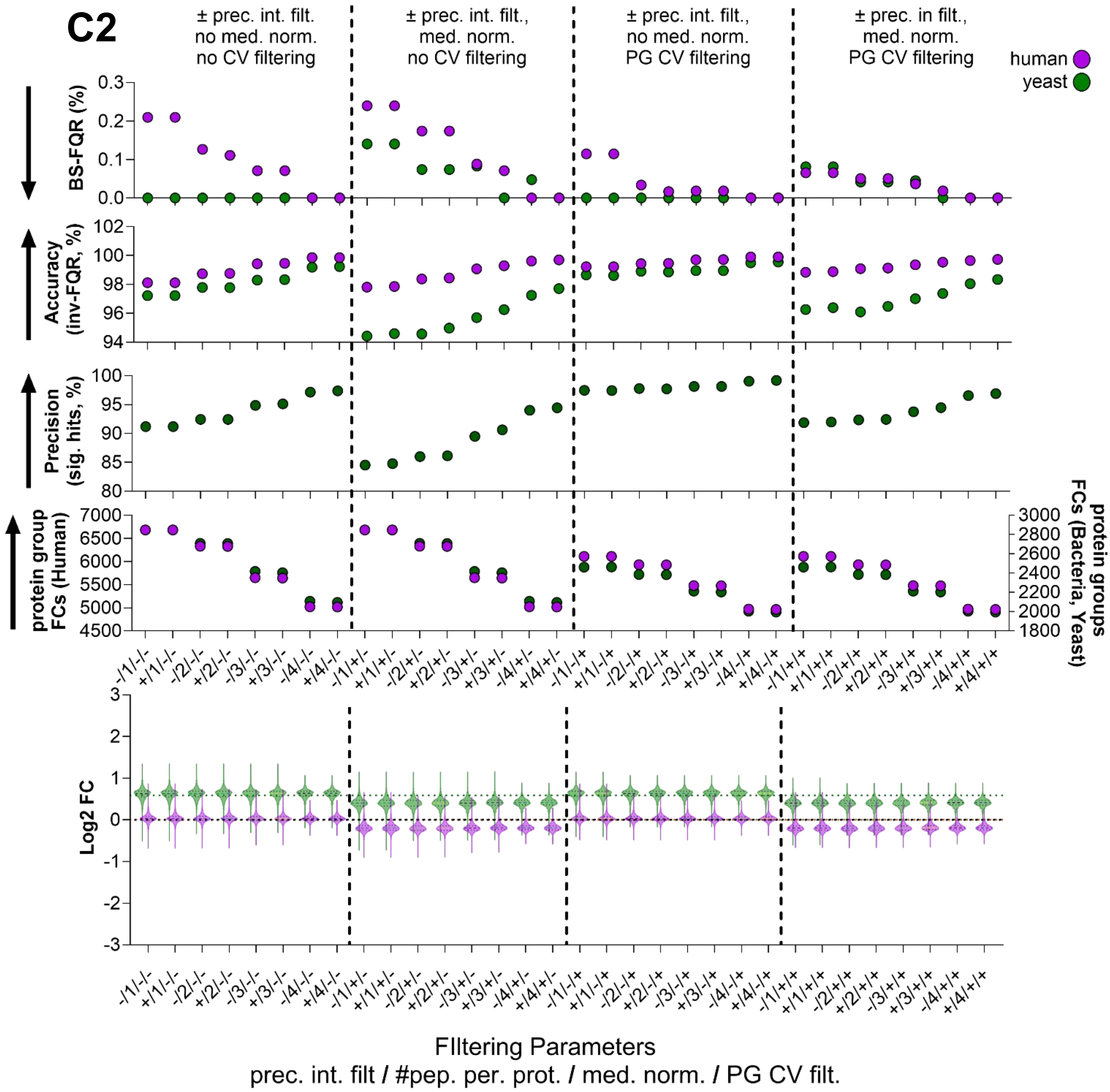
C2
± prec. int. filt.
no med. norm.
no CV filtering
± prec. int. filt.,
med. norm.
no CV filtering
± prec. int. filt.,
no med. norm.
PG CV filtering
± prec. in filt.,
med. norm.
PG CV filtering
human
yeast
BS-FQR (%)
Accuracy (inv-FQR, %)
Precision (sig. hits, %)
protein group FCs (Human)
protein groups FCs (Bacteria, Yeast)
Log2 FC
-/1/-/- +/1/-/- -/2/-/- +/2/-/- -/3/-/- +/3/-/- -/4/-/- +/4/-/- -/1/+/- +/1/+/- -/2/+/- +/2/+/- -/3/+/- +/3/+/- -/4/+/- +/4/+/- -/1/-/+ +/1/-/+ -/2/-/+ +/2/-/+ -/3/-/+ +/3/-/+ -/4/-/+ +/4/-/+ -/1/+/+ +/1/+/+ -/2/+/+ +/2/+/+ -/3/+/+ +/3/+/+ -/4/+/+ +/4/+/+
FIltering Parameters
prec. int. filt / #pep. per. prot. / med. norm. / PG CV filt.

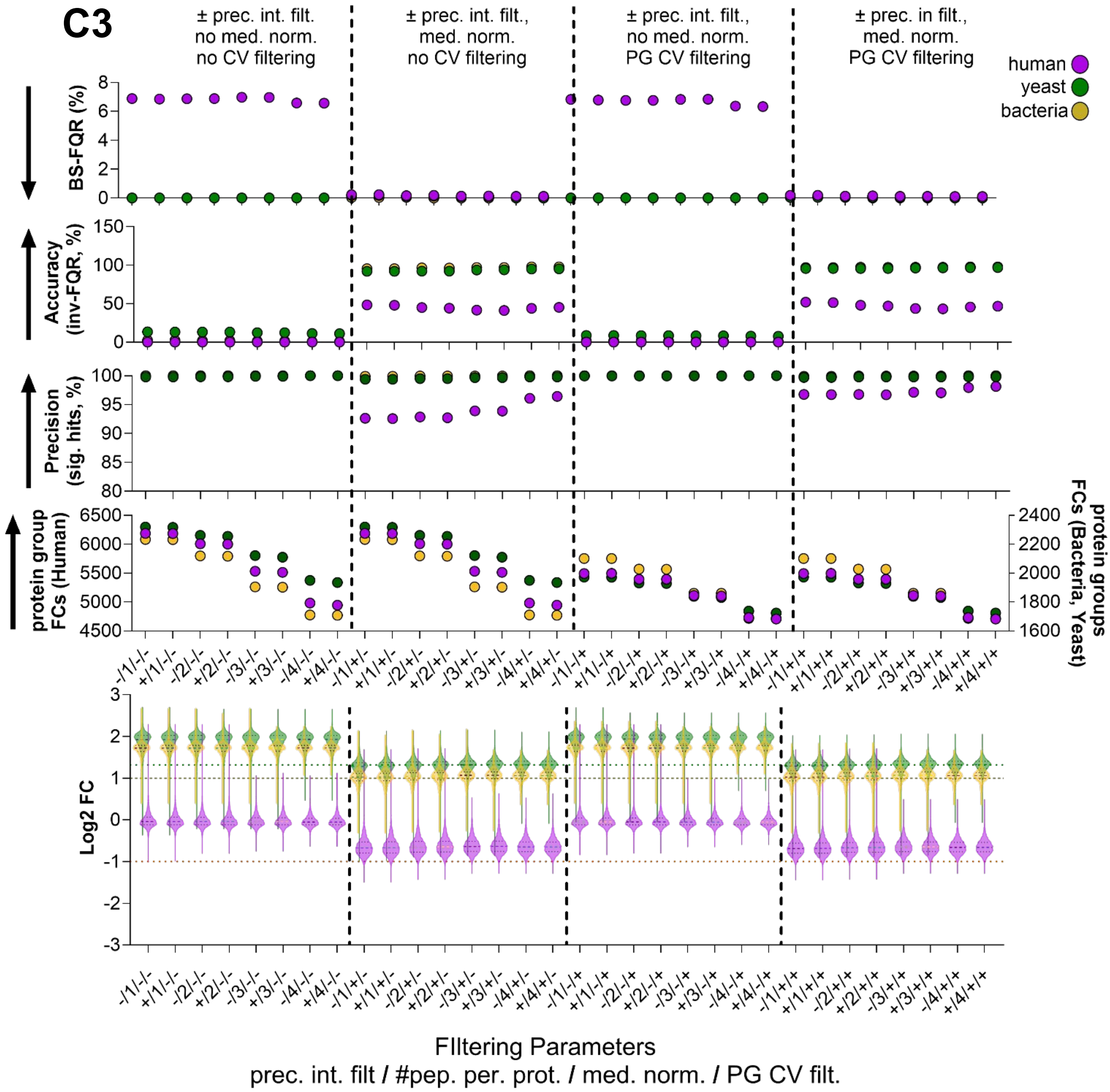
C3
± prec. int. filt. no med. norm. no CV filtering
± prec. int. filt., med. norm. no CV filtering
± prec. int. filt., no med. norm. PG CV filtering
± prec. in filt., med. norm. PG CV filtering
human
yeast
bacteria
BS-FQR (%)
Accuracy (inv-FQR, %)
Precision (sig. hits, %)
protein group FCs (Human)
protein groups FCs (Bacteria, Yeast)
Log2 FC
FIltering Parameters
prec. int. filt / #pep. per. prot. / med. norm. / PG CV filt.

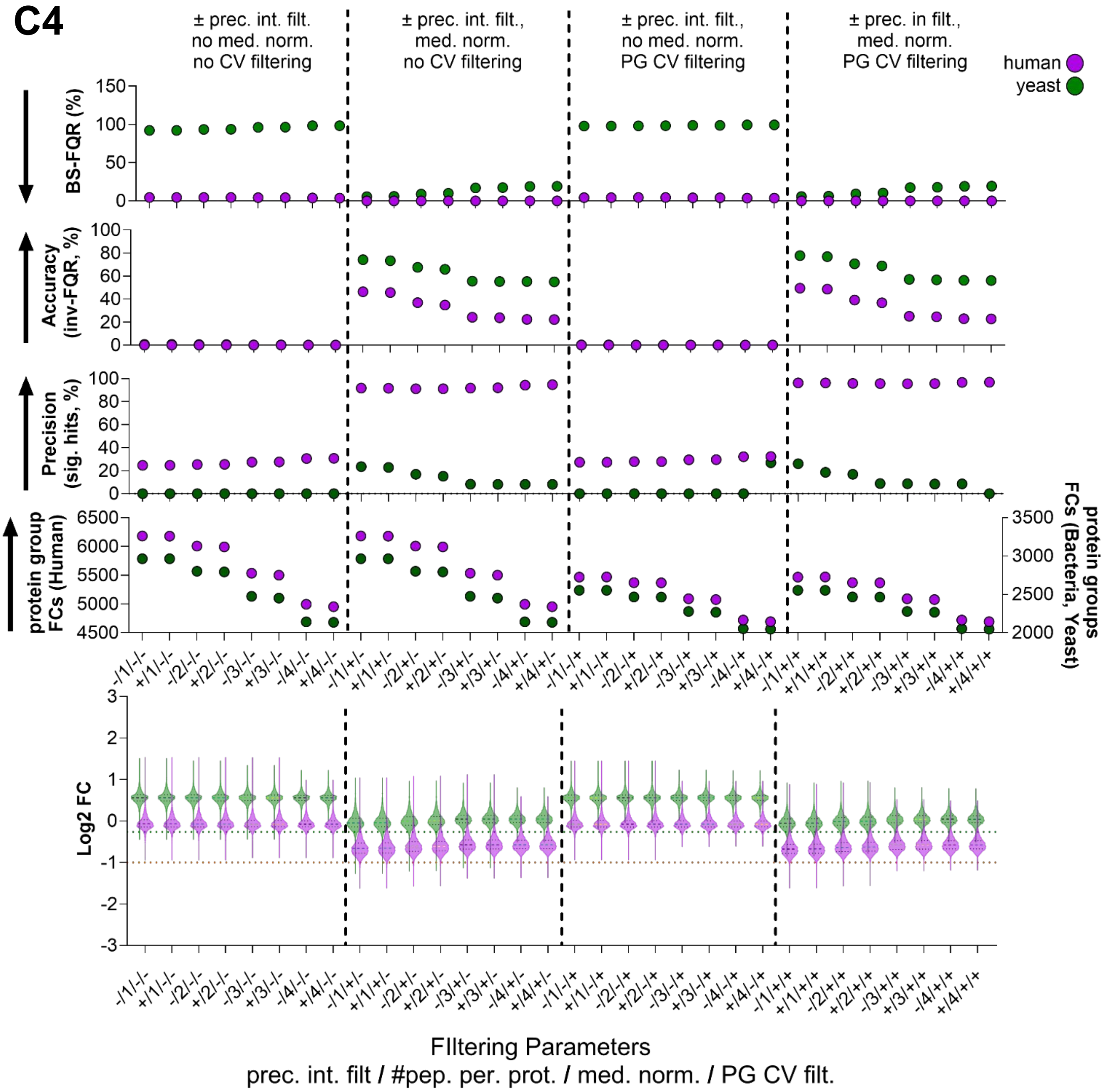
C4
± prec. int. filt.
no med. norm.
no CV filtering
± prec. int. filt.,
med. norm.
no CV filtering
± prec. int. filt.,
no med. norm.
PG CV filtering
± prec. in filt.,
med. norm.
PG CV filtering
human
yeast
BS-FQR (%)
Accuracy (inv-FQR, %)
Precision (sig. hits, %)
protein group FCs (Human)
protein groups FCs (Bacteria, Yeast)
Log2 FC
-/1/-/- +/1/-/- -/2/-/- +/2/-/- -/3/-/- +/3/-/- -/4/-/- +/4/-/- -/1/+/- +/1/+/- -/2/+/- +/2/+/- -/3/+/- +/3/+/- -/4/+/- +/4/+/- -/1/-/+ +/1/-/+ -/2/-/+ +/2/-/+ -/3/-/+ +/3/-/+ -/4/-/+ +/4/-/+ -/1/+/+ +/1/+/+ -/2/+/+ +/2/+/+ -/3/+/+ +/3/+/+ -/4/+/+ +/4/+/+
FIltering Parameters
prec. int. filt / #pep. per. prot. / med. norm. / PG CV filt.

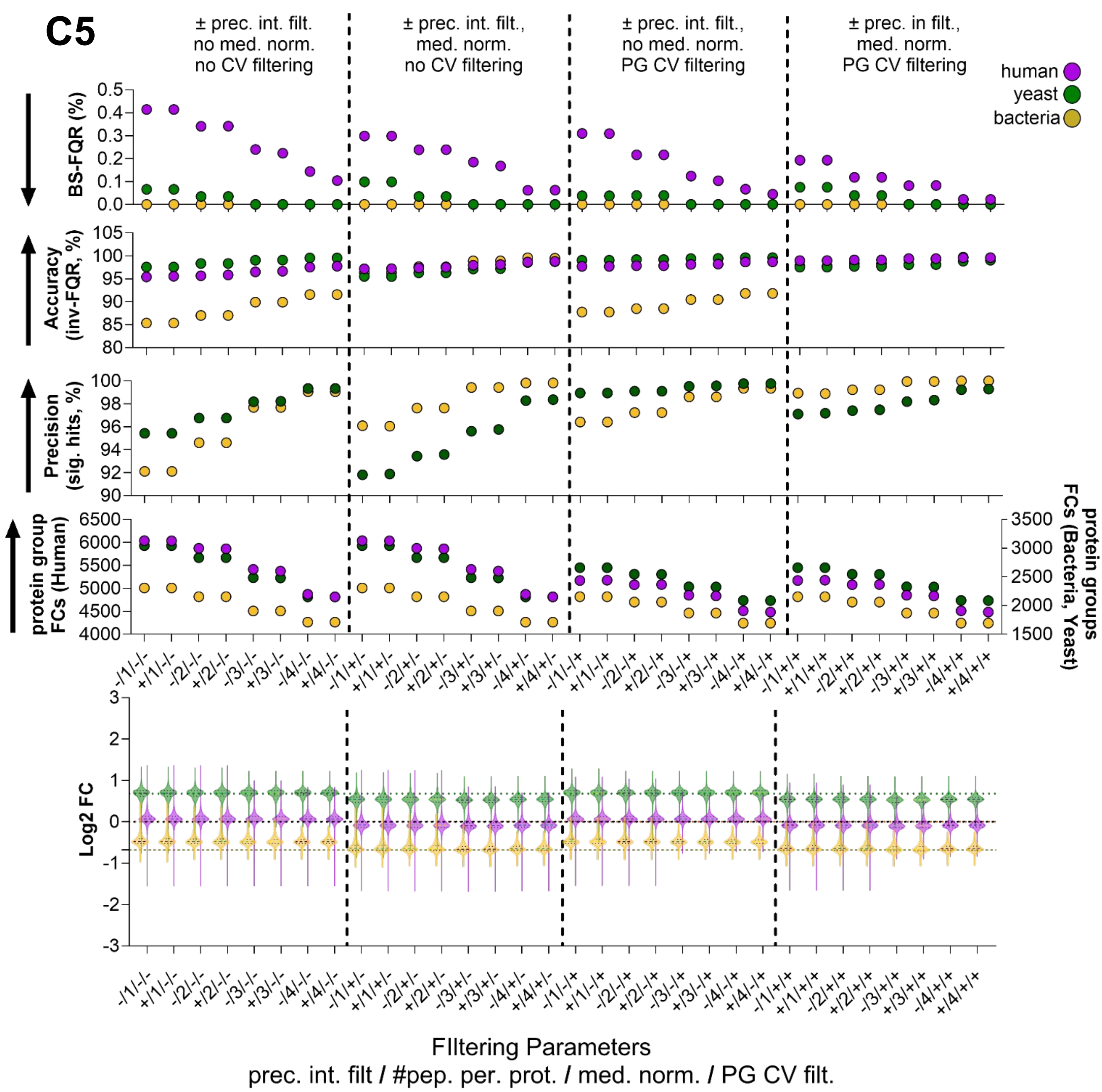
C5
± prec. int. filt. no med. norm. no CV filtering
± prec. int. filt., med. norm. no CV filtering
± prec. int. filt., no med. norm. PG CV filtering
± prec. in filt., med. norm. PG CV filtering
human
yeast
bacteria
BS-FQR (%)
Accuracy (inv-FQR, %)
Precision (sig. hits, %)
protein group FCs (Human)
protein groups FCs (Bacteria, Yeast)
Log2 FC
FIltering Parameters
prec. int. filt / #pep. per. prot. / med. norm. / PG CV filt.

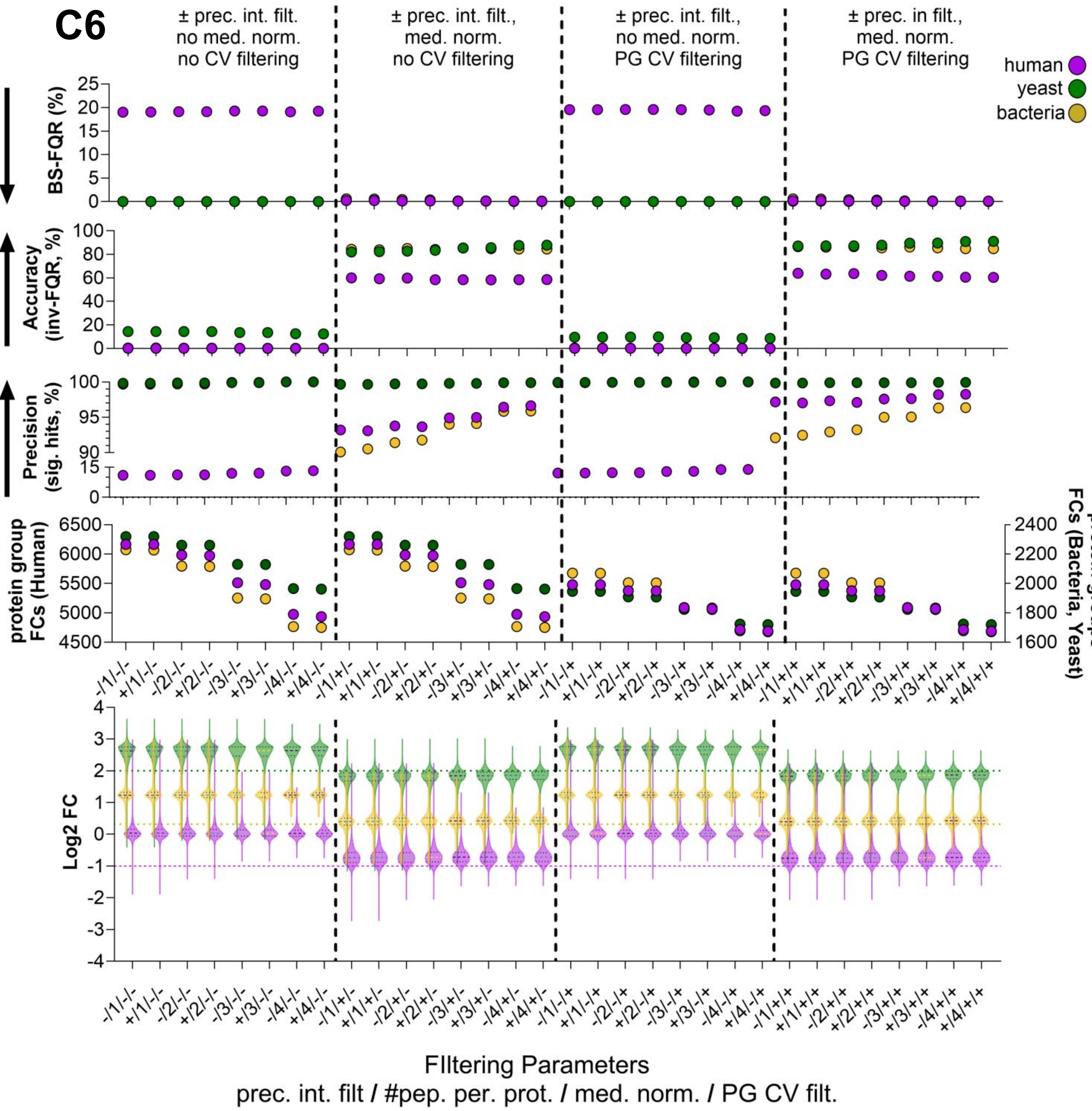

Figure S3. Post processing impacts quantitative efficacy in typical and atypical proteome comparisons derived from timsToF-HT analyses. Purple = Human, Green= Yeast Yellow = Bacteria. Data shown are derived from five replicates. The comparisons (C1-C6) are indicated. Arrows indicate the preferred vector for each metric. Horizontal lines indicate the expected FC in the Log2FC distribution plot.

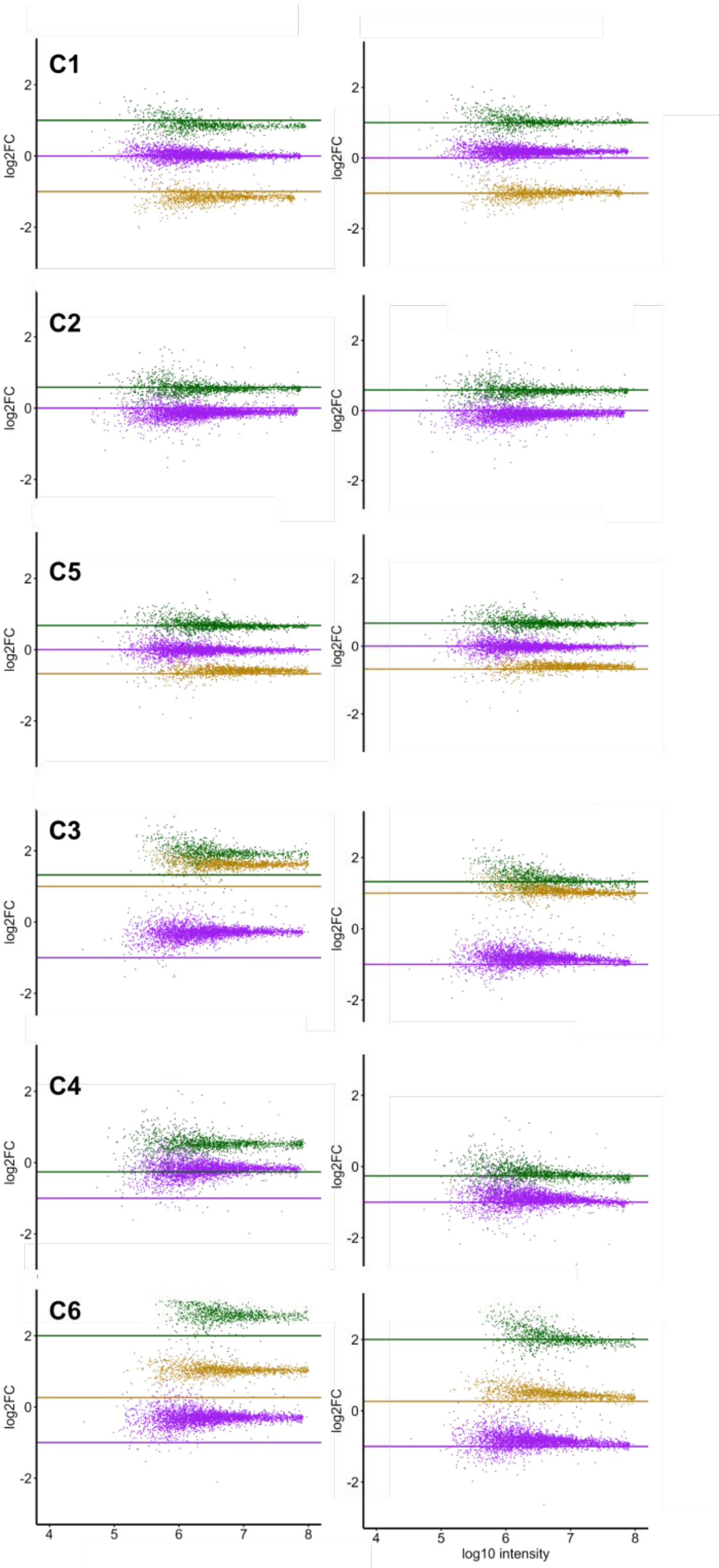

Figure S4. Median normalisation alters distribution of measured protein group FC for E480 data. FC shown here is the default post-processing pipeline (2 peptides per protein, protein present in each condition).

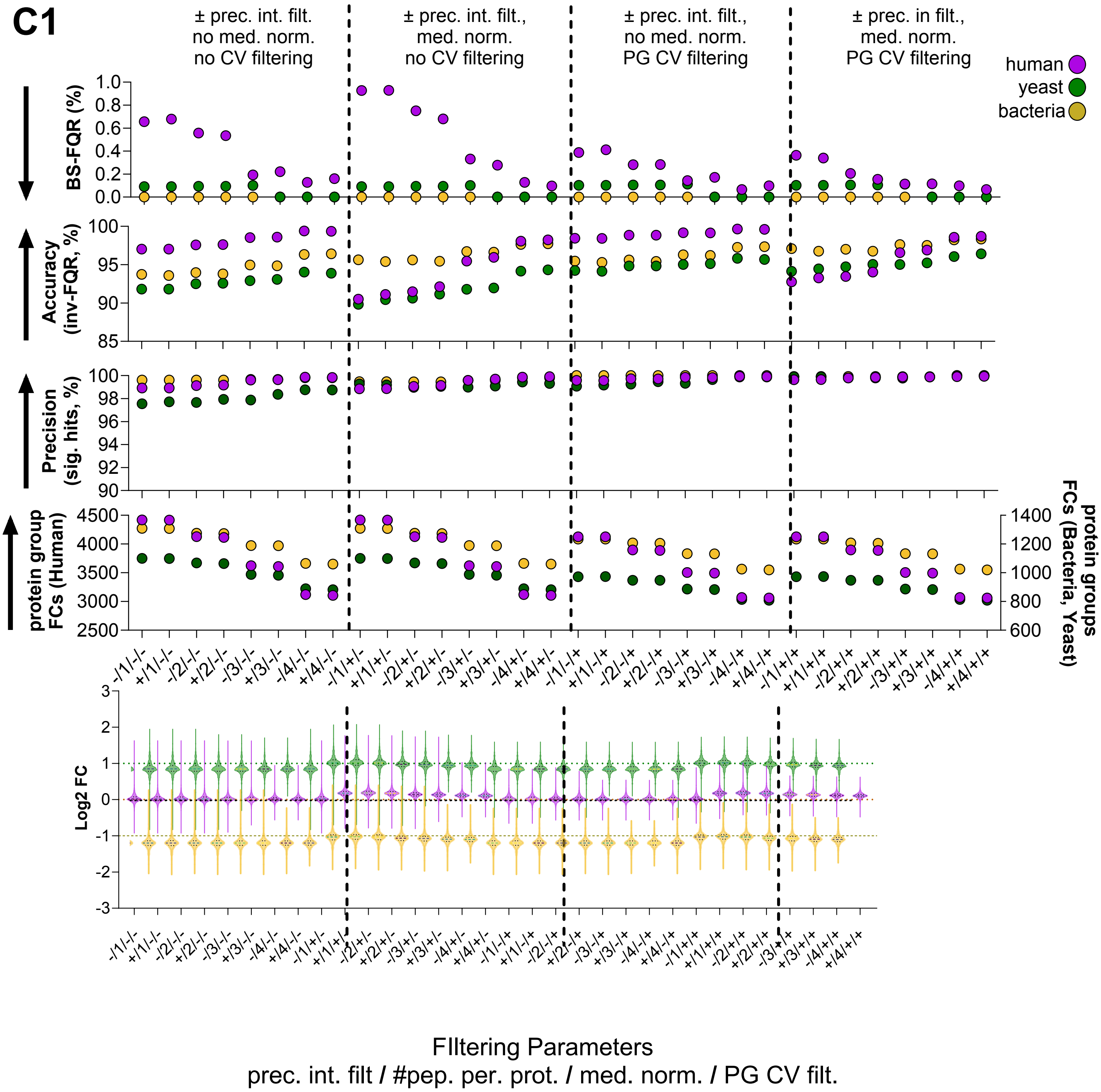
C1
± prec. int. filt.
no med. norm.
no CV filtering
± prec. int. filt.,
med. norm.
no CV filtering
± prec. int. filt.,
no med. norm.
PG CV filtering
± prec. in filt.,
med. norm.
PG CV filtering
human
yeast
bacteria
BS-FQR (%)
Accuracy (inv-FQR, %)
Precision (sig. hits, %)
protein group FCs (Human)
protein groups FCs (Bacteria, Yeast)
Log2 FC
FIltering Parameters
prec. int. filt / #pep. per. prot. / med. norm. / PG CV filt.

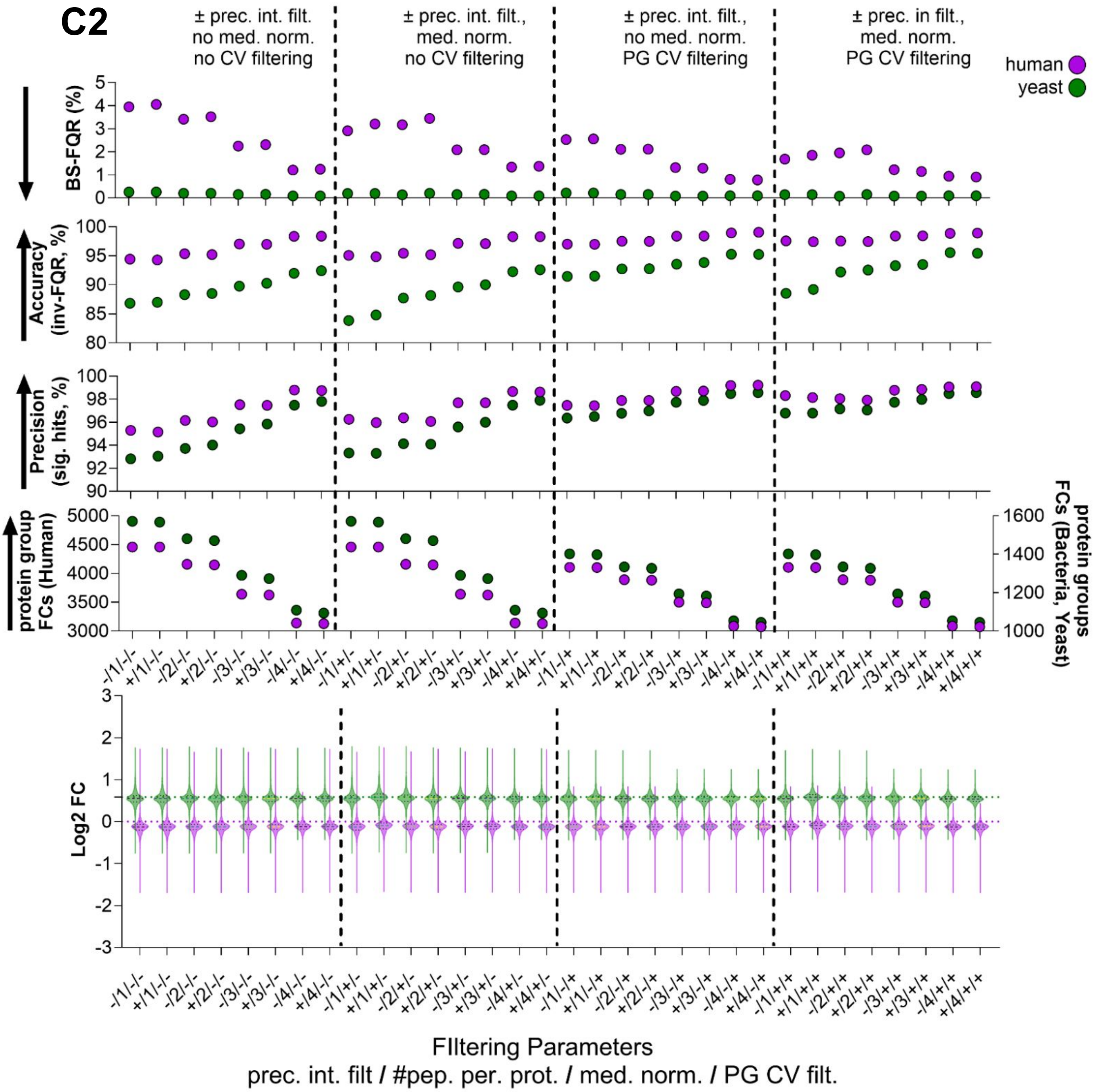
C2
± prec. int. filt. no med. norm. no CV filtering
± prec. int. filt., med. norm. no CV filtering
± prec. int. filt., no med. norm. PG CV filtering
± prec. in filt., med. norm. PG CV filtering
human
yeast
BS-FQR (%)
Accuracy (inv-FQR, %)
Precision (sig. hits, %)
protein group FCs (Human)
protein groups FCs (Bacteria, Yeast)
Log2 FC
FIltering Parameters
prec. int. filt / #pep. per. prot. / med. norm. / PG CV filt.

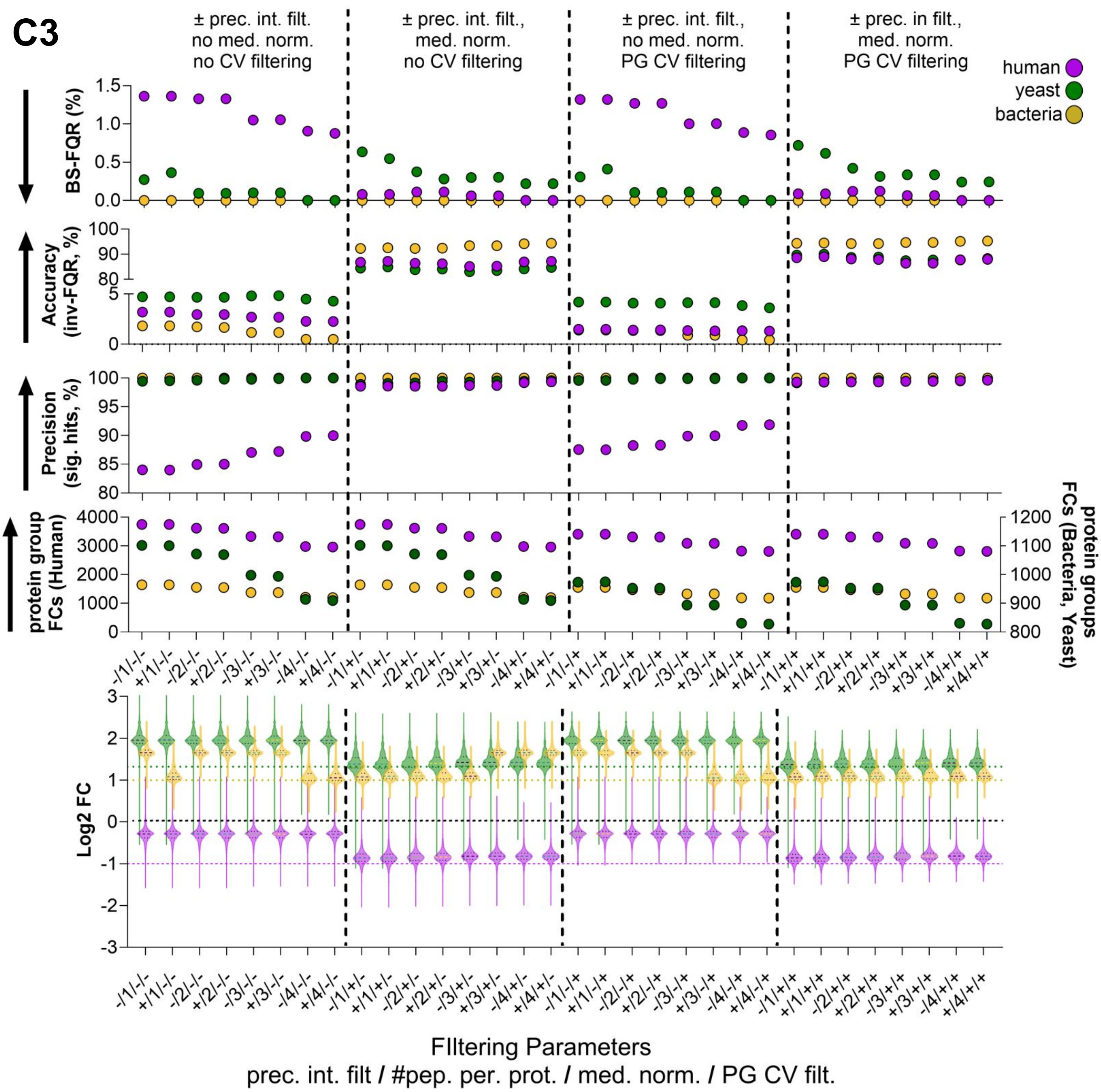
C3
± prec. int. filt. no med. norm. no CV filtering
± prec. int. filt., med. norm. no CV filtering
± prec. int. filt., no med. norm. PG CV filtering
± prec. in filt., med. norm. PG CV filtering
human
yeast
bacteria
BS-FQR (%)
Accuracy (inv-FQR, %)
Precision (sig. hits, %)
protein group FCs (Human)
protein groups FCs (Bacteria, Yeast)
Log2 FC
FIltering Parameters
prec. int. filt / #pep. per. prot. / med. norm. / PG CV filt.

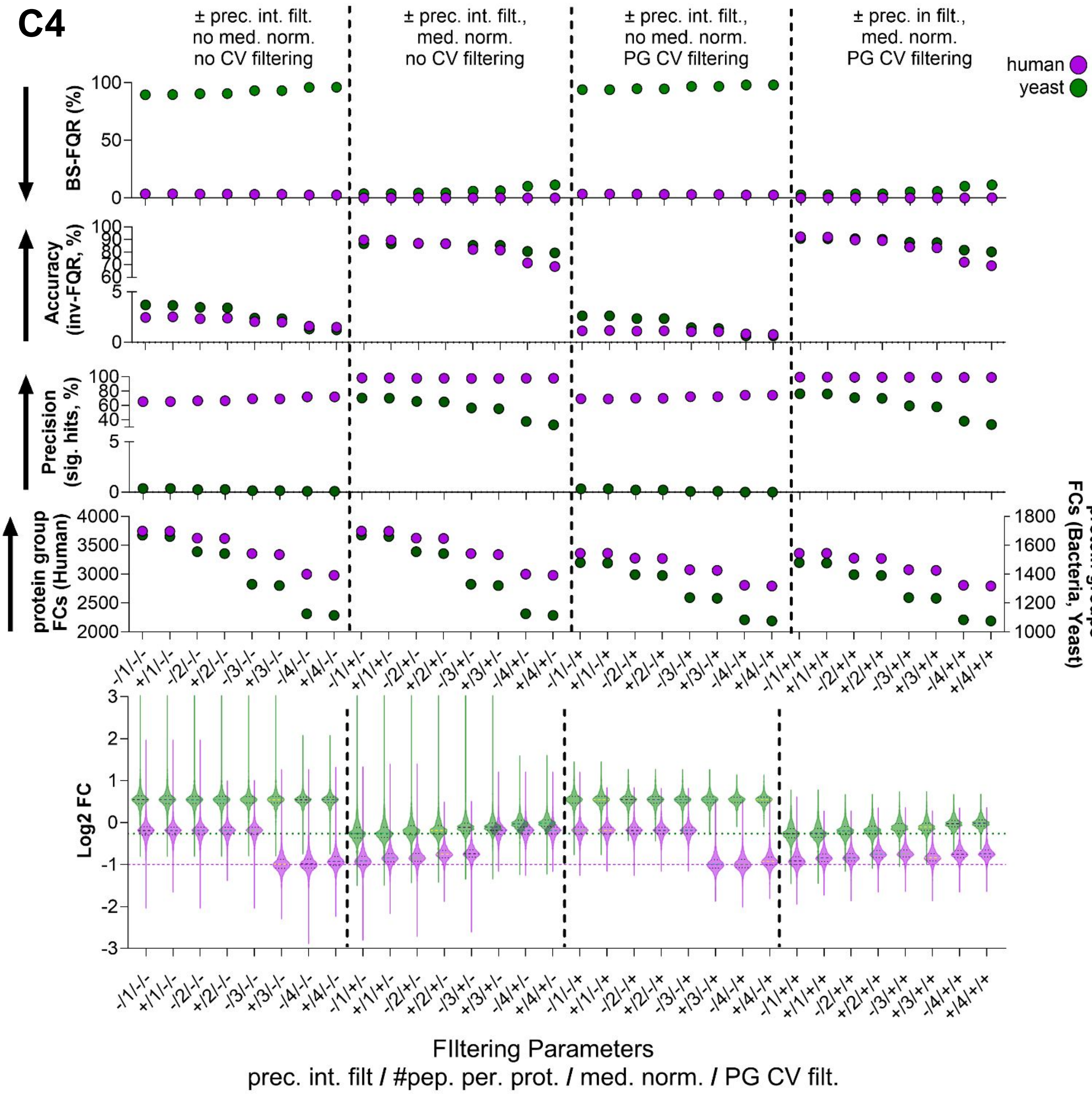
C4
± prec. int. filt. no med. norm. no CV filtering
± prec. int. filt., med. norm. no CV filtering
± prec. int. filt., no med. norm. PG CV filtering
± prec. in filt., med. norm. PG CV filtering
human
yeast
BS-FQR (%)
Accuracy (inv-FQR, %)
Precision (sig. hits, %)
protein group FCs (Human)
protein groups FCs (Bacteria, Yeast)
Log2 FC
FIltering Parameters
prec. int. filt / #pep. per. prot. / med. norm. / PG CV filt.

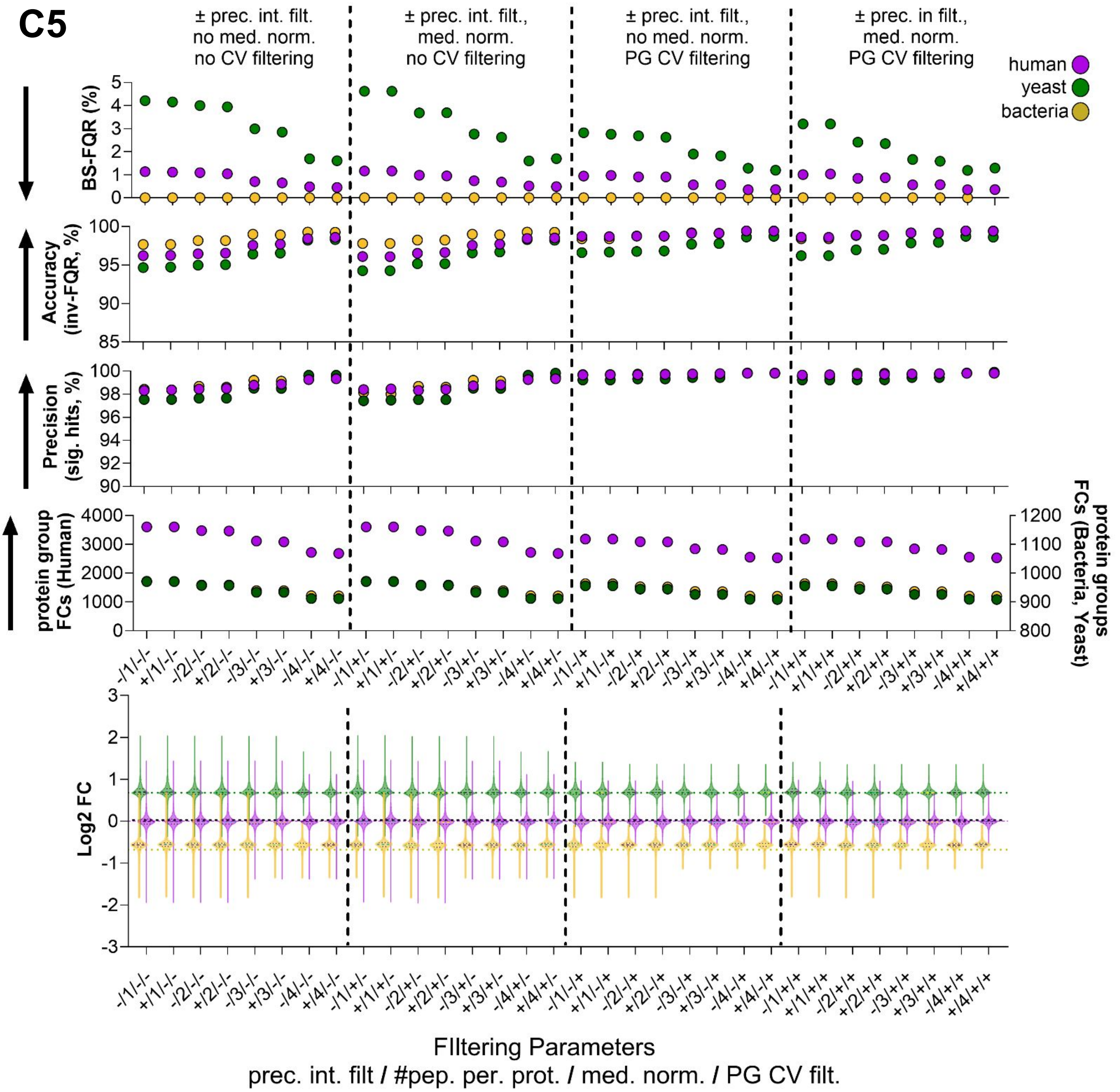
C5
± prec. int. filt.
no med. norm.
no CV filtering
± prec. int. filt.,
med. norm.
no CV filtering
± prec. int. filt.,
no med. norm.
PG CV filtering
± prec. in filt.,
med. norm.
PG CV filtering
human
yeast
bacteria
BS-FQR (%)
Accuracy (inv-FQR, %)
Precision (sig. hits, %)
protein group FCs (Human)
protein groups FCs (Bacteria, Yeast)
Log2 FC
FIltering Parameters
prec. int. filt / #pep. per. prot. / med. norm. / PG CV filt.

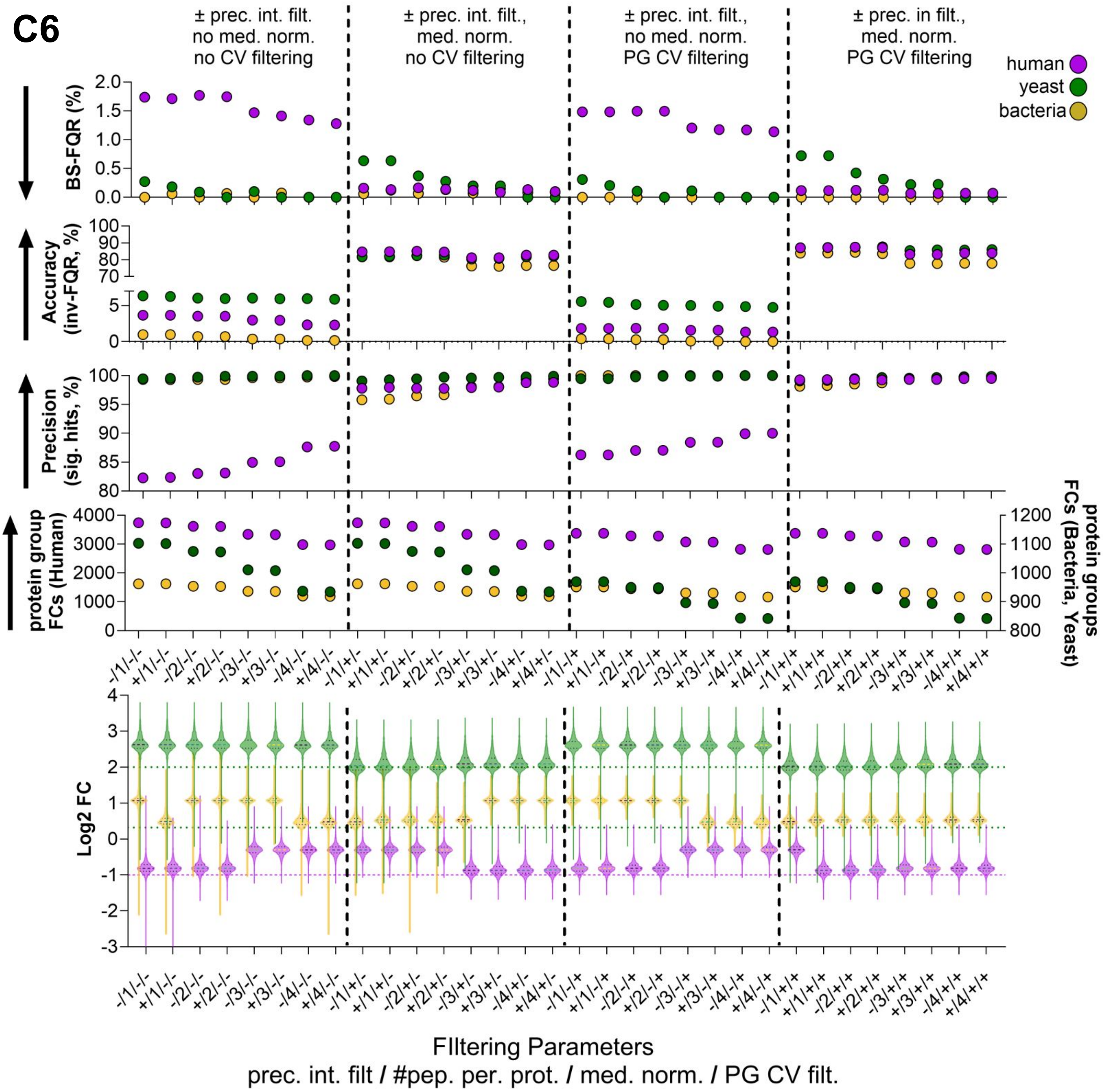

Figure S5. Post processing impacts quantitative efficacy in typical and atypical proteome comparisons derived from Orbitrap Exploris 480 analyses. Purple = Human, Green= Yeast Yellow = Bacteria. Data shown are derived from five replicates. The comparisons (C1-C6) are indicated. Arrows indicate the preferred vector for each metric. Horizontal lines indicate the expected FC in the Log2FC distribution plot.

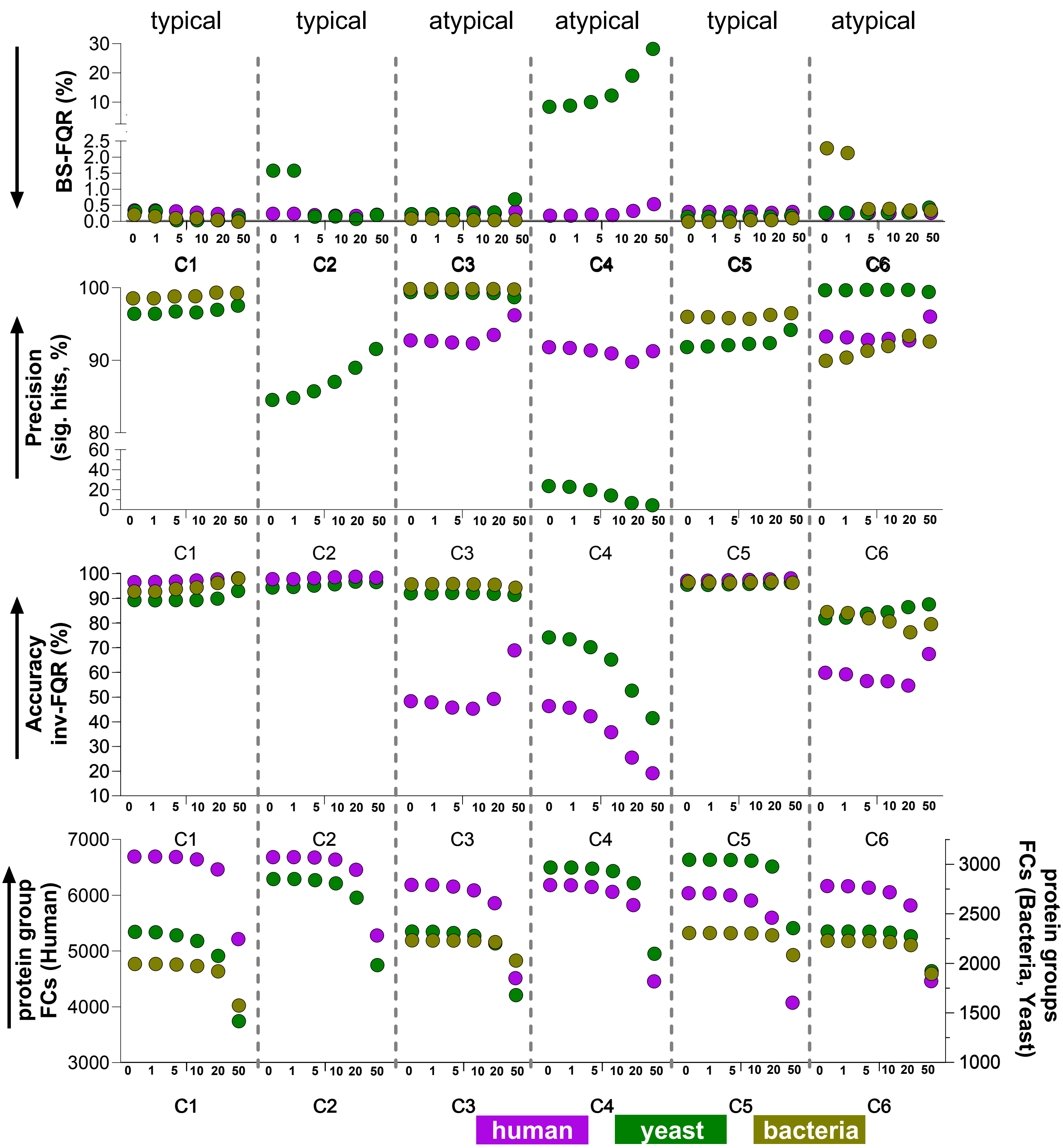

Figure S6. Precursor intensity filtering impacts quantitative efficacy in typical and atypical proteome comparisons derived from timsToF-HT analyses. Data shown are derived from five replicates. The comparisons (C1-C6) are indicated. Arrows indicate the preferred vector for each metric. X axis numbers indicate mean precursor intensity filtering, different percentiles were excluded, either zero filtering (0 %), or <1, <10, <20, or <50 % were excluded prior to protein group quantification.

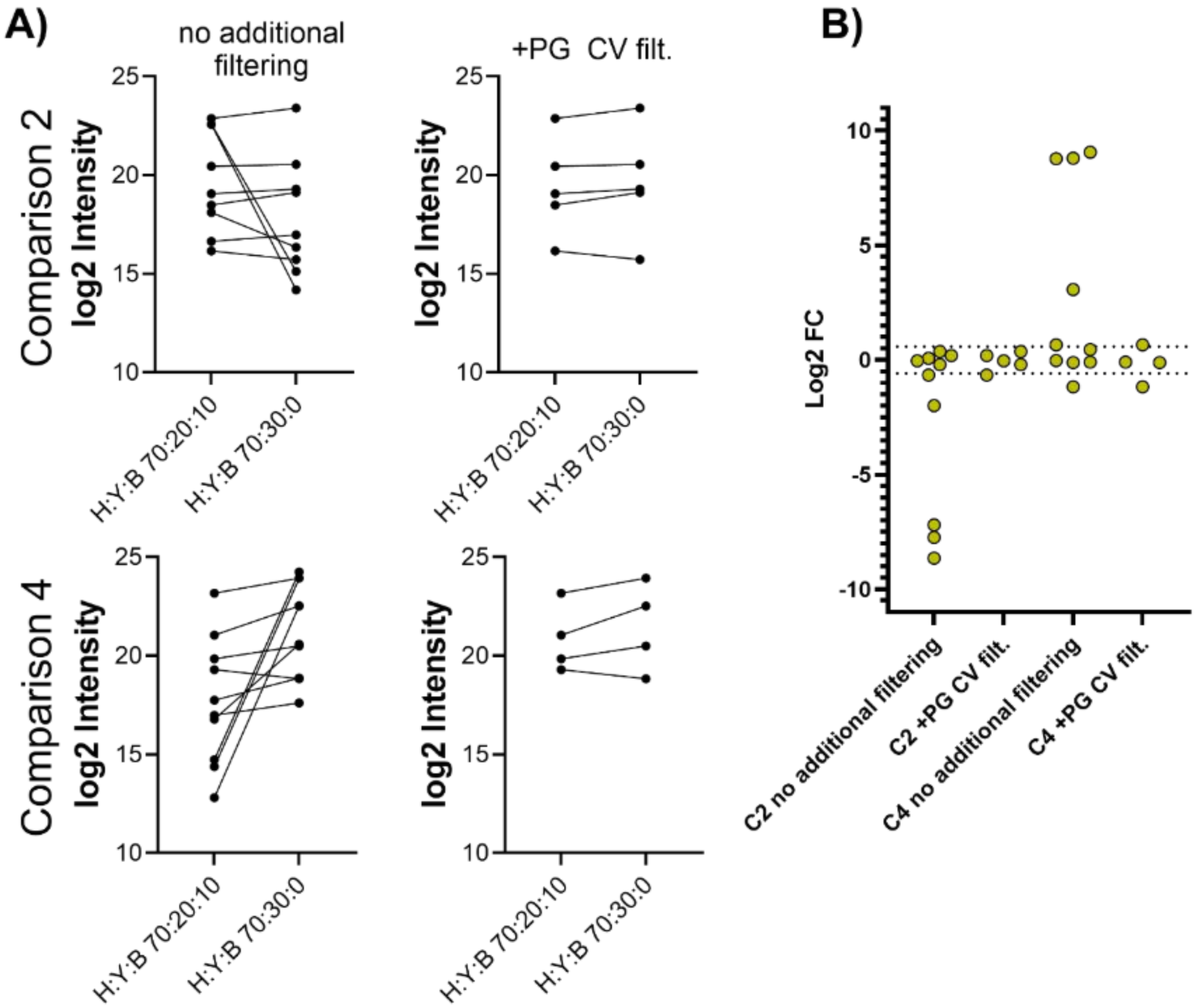

Figure S7. Falsely-discovered changes in relative abundance are minimal, but may be present in typical proteomics analyses. A) Intensity of the individual *E. coli* proteins quantified in comparisons 2 and 4, where one condition lacks *E. coli*. B) Log2 FC of *E. coli* proteins. Dotted lines indicate ±1.5-fold change. Data undergo either minimal post processing, or +protein group CV filtering as indicated. Data are derived from 5 replicates.

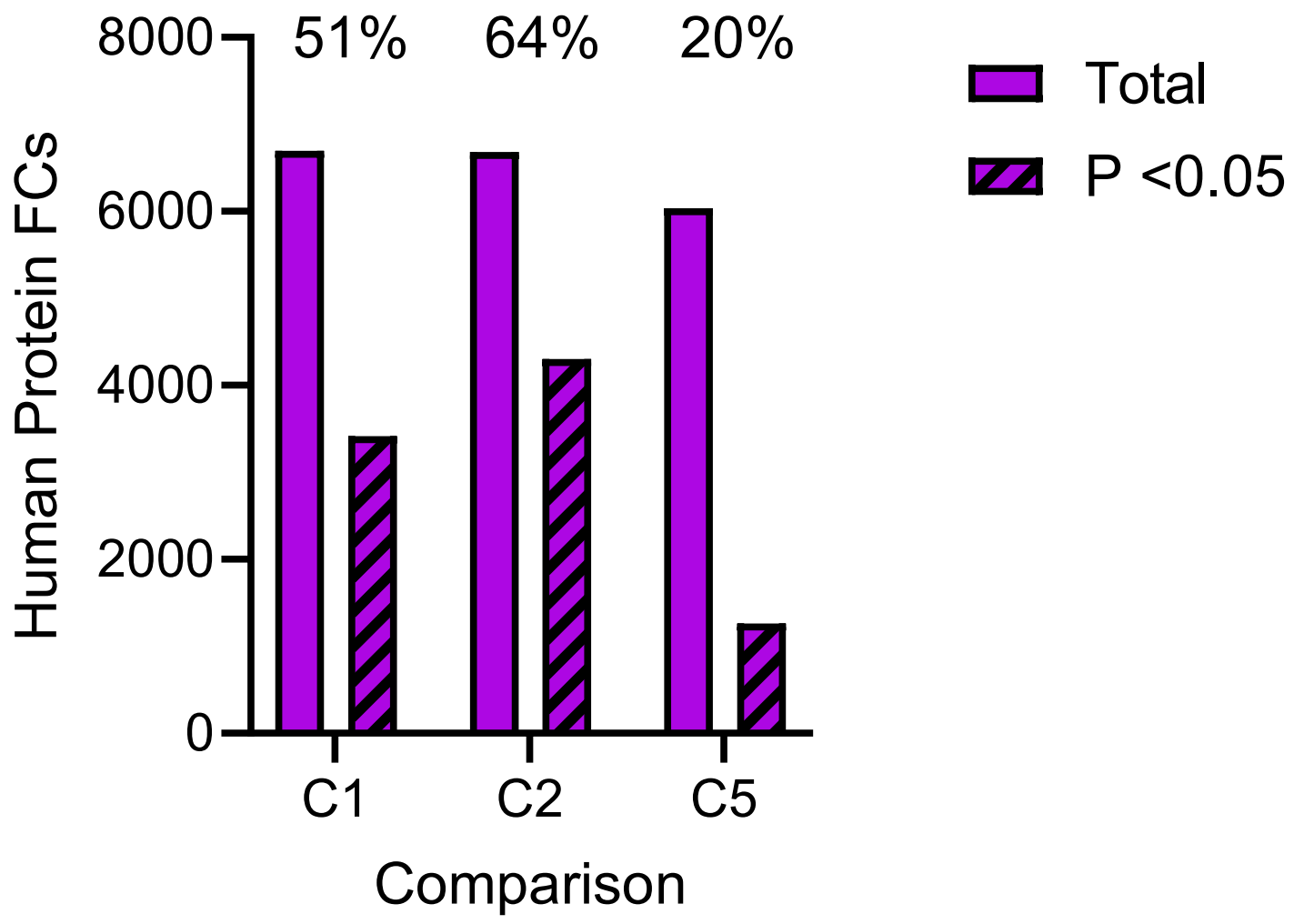

Figure S8. Human proteins and those with statistical significance in comparisons with no ground-truth abundance change. The number of human proteins relatively quantified in typical comparisons (C1, C2, C5) from timsToF experiments with minimal filtering are indicated, along with the number of these determined to be statistically significant (T-test with Benjamini Hochberg correction in Limma, adjusted P=<0.05). Percentages indicate the proportion of statistically significant proteins.

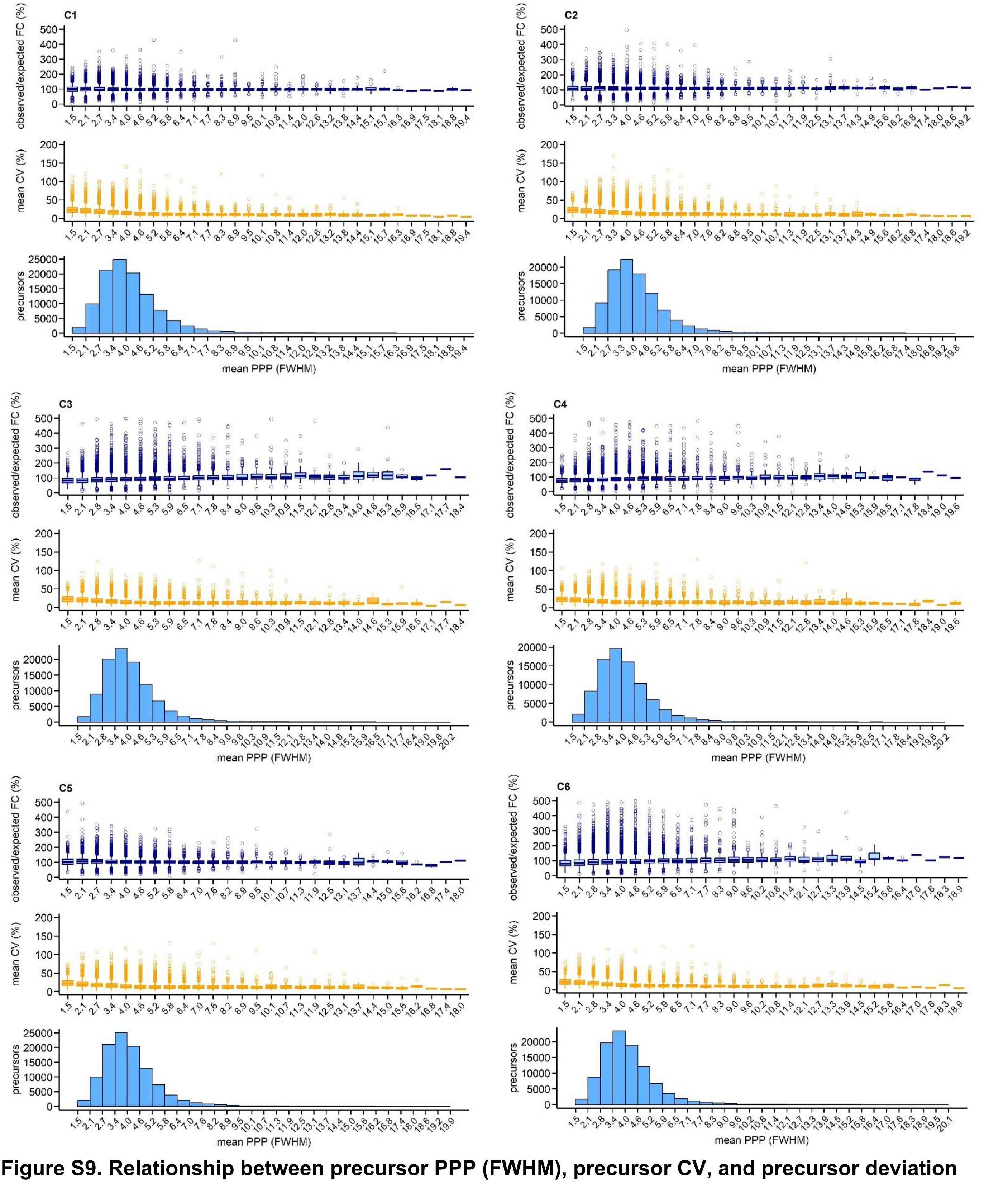

Figure S9. Relationship between precursor PPP (FWHM), precursor CV, and precursor deviation from ground truth. timsToF precursors (with no filtering) were placed into 30 bins based on their PPP, for each comparison. For each precursor bin, the number of precursors was plotted-lowest histograms, as was the mean CV (%) and deviation from ground truth (observed/expected fold change, %)-middle and upper boxplots. Boxplots show median, quartiles, outliers. Data derived from 5 replicates.

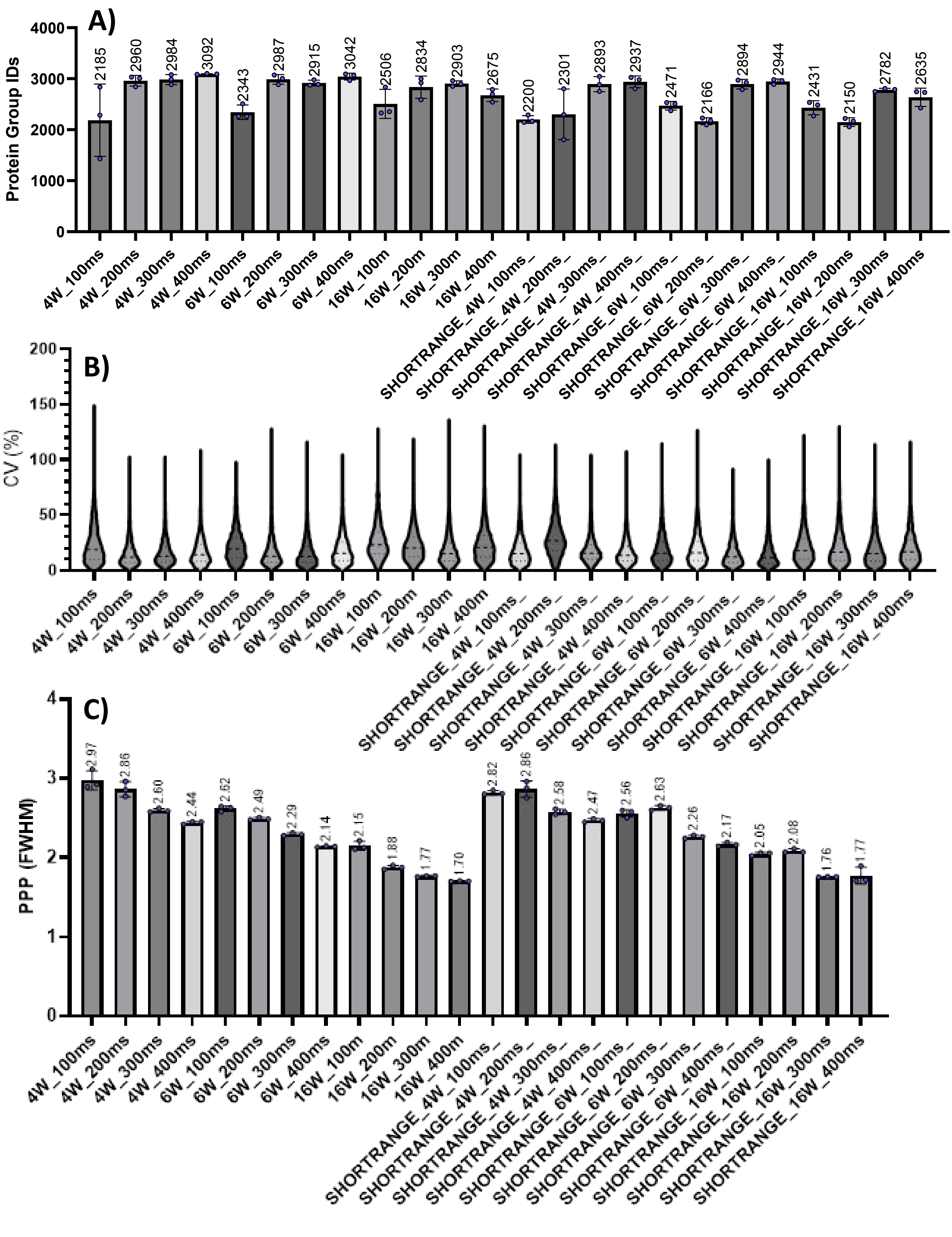

Figure S10. High sensitivity diaPASEF optimisation-ID rates and reproducibility for single-cell level loads on the timsToF HT. Tryptic HeLa digest-250pg-was subjected to different TARTs, PASEF window numbers, and mass ranges (*m/z* 300-1200 was considered default and not labeled, *m/z* 450-950 was short range). A) Protein group IDs, mean (bars) and replicate values (circles) shown. Error bars = SD. B) CV violin plot, dashed lines indicate median, and upper and lower quartiles. C) Points per peak (full width at half maximum), mean (bars) and replicate values (circles) shown. Error bars = SD.
